# Cell-Penetrating Dabcyl-Containing Tetraarginines with Backbone Aromatics as Uptake Enhancers

**DOI:** 10.3390/pharmaceutics15010141

**Published:** 2022-12-31

**Authors:** Mo’ath Yousef, Ildikó Szabó, József Murányi, Françoise Illien, Dóra Soltész, Csaba Bató, Gabriella Tóth, Gyula Batta, Péter Nagy, Sandrine Sagan, Zoltán Bánóczi

**Affiliations:** 1 Department of Organic Chemistry, Eötvös L. University, 1117 Budapest, Hungary; 2 MTA-ELTE Research Group of Peptide Chemistry, Eötvös Loránd Research Network (ELKH), 1117 Budapest, Hungary; 3 Department of Molecular Biology, Institute of Biochemistry and Molecular Biology, Semmelweis University, 1094 Budapest, Hungary; 4 MTA-SE Pathobiochemistry Research Group, 1094 Budapest, Hungary; 5 Sorbonne Université, École Normale Supérieure, PSL University, CNRS, Laboratoire des Biomolécules, LBM, 75005 Paris, France; 6 Department of Biophysics and Cell Biology, Faculty of Medicine, University of Debrecen, 4032 Debrecen, Hungary; 7 Department of Genetics and Applied Microbiology, Faculty of Science and Technology, University of Debrecen, 4032 Debrecen, Hungary

**Keywords:** cell-penetrating peptide, drug delivery, peptide engineering, cellular internalization

## Abstract

Cell-penetrating peptides represent an emerging class of carriers capable of effective cellular delivery. This work demonstrates the preparation and investigation of efficient CPPs. We have already shown that the presence of 4-((4-(dimethylamino)phenyl)azo)benzoic acid (Dabcyl) and Trp greatly increase the uptake of oligoarginines. This work is a further step in that direction. We have explored the possibility of employing unnatural, aromatic amino acids, to mimic Trp properties and effects. The added residues allow the introduction of aromaticity, not as a side-chain group, but rather as a part of the sequence. The constructs presented exceptional internalization on various cell lines, with an evident structure–activity relationship. The CPPs were investigated for their entry mechanisms, and our peptides exploit favorable pathways, yet one of the peptides relies highly on direct penetration. Confocal microscopy studies have shown selectivity towards the cell lines, by showing diffuse uptake in FADU cells, while vesicular uptake takes place in SCC-25 cell line. These highly active CPPs have proved their applicability in cargo delivery by successfully delivering antitumor drugs into MCF-7 and MDA-MB-231 cells. The modifications in the sequences allow the preparation of short yet highly effective constructs able to rival the penetration of well-known CPPs such as octaarginine (Arg8).

## 1. Introduction

The delivery of biologically active molecules across the cellular membrane is often challenging, since cellular membranes prevent the internalization of many molecules into cells. The inefficient uptake of molecules necessitates the need for an effective delivery system able to provide additional pathways for cellular entry. Cell-penetrating peptides (CPPs) are transport vectors that can tackle the problem of poor cellular delivery. Such vehicles were shown to efficiently deliver small drug molecules [1,2,3,4,5,6], peptides [7,8], and large biomolecules, such as proteins [9]. While CPPs deliver their cargo effectively, they are able to cross the cellular membrane without inducing high cytotoxicity [10,11,12].

The penetrative nature of specific proteins was first demonstrated in 1988, by the trans-activator of transcription (TAT), derived from human immunodeficiency virus (HIV-1). TAT was able to cross the cellular membrane effectively. It was later discovered that the sequence GRKKRRQRRRPPQ, termed TAT48-60, was responsible for this [13,14]. Another peptide showing similar properties is penetratin (RQIKIWFQNRRMKWKK), which was derived from the Drosophila Antennapedia homeodomain [15].

Both the TAT48-60 and penetratin contain many arginine residues and it was later pointed out that Arg is largely responsible for the high uptake of TAT48-60 [16] due its charged guanidinium group in the side chain [17]. This led to the synthesis of CPPs that contain mainly Arg residues, such as octaarginine (Arg_8_) [18].

Oligoarginines (Arg_8-12_) are effective CPPs, but further modifications can improve their internalization [19]. One such modification can be the addition of tryptophan. Replacement of proline (Pro) with tryptophan (Trp) resulted in increased uptake of Tat48-60 [20], while replacing Trp with Leucine (Leu) diminished the uptake [21]. As Trp positively affects the uptake of CPPs, its role has been extensively studied, especially its position and quantity within a sequence [10,22,23,24,25,26,27,28]. 

Another modifier to oligoarginines, was the inclusion of a hydrophobic moiety at the N-terminus. We have reported that coupling of 4-((4-(dimethylamino)phenyl)azo)benzoic acid (Dabcyl), a well-known quencher in FRET pairs [29,30,31], to oligoarginines increases the uptake dramatically [32]. This was a significant result, since CPPs with less than eight Arg residues are generally deemed ineffective [16]. Recently, we extended our approach to include Trp into the sequence of Dabcyl-containing tetraarginines. The presence of Dabcyl and Trp further increased the uptake of tetraarginines, where the structure directly impacted the activity [33].

While Trp can have a positive influence on the uptake of oligoarginines, other unnatural aromatic moieties may have a similar effect, and thus influence activity. In this work, we combine the use of Dabcyl with the unnatural residues 4-aminobenzoic acid (PABA), 4-(aminomethyl) benzoic acid (AMBA), and 6-amino-2-naphthoic acid (NAPH). We assumed that the presence of these aromatic residues can indeed mimic Trp, endowing short polyarginine sequences with increased uptake activity. 

## 2. Results

### 2.1. Synthesis of Peptides

In Arg-rich peptides, the increasing number of Arg residues correlates with the enhanced uptake, where 8–10 Arg residues are the optimum [34]. In other peptides, not only Arg, but the presence of Trp is also essential for effective internalization. For example, replacing it with phenylalanine (Phe) diminishes the uptake of penetratin [35]. This has prompted the synthesis of Trp-containing, Arg-rich CPPs with improved internalization property [10,26,36]. We described that Dabcyl group increases the cellular uptake of hexa- and tetraarginine [32] and its effect may be further enhanced by Trp [33]. Two tetraarginine derivatives (Dabcyl-RRWRRK and Dabcyl-WRRRRK) were proven to be effective. 

**Figure 1:**
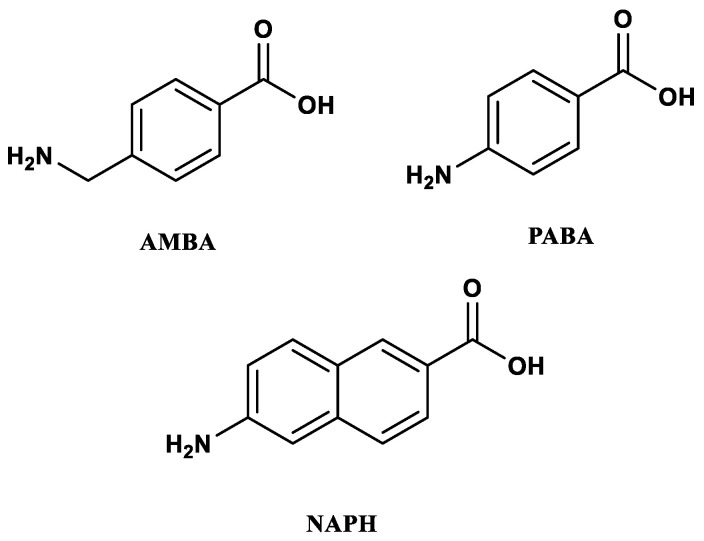
Structures of the used non-natural aromatic amino acids, AMBA, PABA and NAPH.

**Scheme 1:**
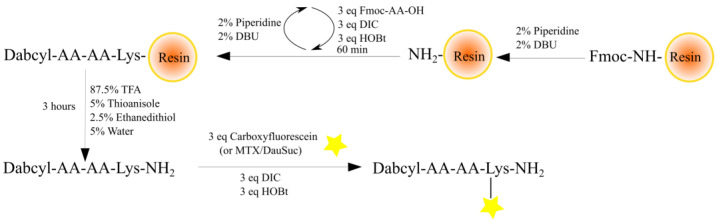
Schematic diagram of the synthesis of peptides.

In this work, three non-natural amino acids were selected to replace the Trp in the sequence of tetraarginine derivatives (Dabcyl-RRWRRK and Dabcyl-WRRRRK) (Figure 1). The common property of their usage is that the aromatic ring(s) is built into the peptide backbone and not in the side chain. Based on our knowledge, this is a novel modification of cell-penetrating peptides, even of peptides. The insertion of PABA and NAPH may result in more rigidity in the peptide backbone while the AMBA is a more flexible system, because of the methylene group between the aromatic ring and amino group. All peptides were synthesized manually by Fmoc/tBu strategy. For the coupling of amino acids, DIC and HOBt were used, but the next amino acids after NAPH and PABA were coupled using HATU (Scheme 1). To study the uptake of peptides fluorescent molecules, (5(6)-carboxyfluorescein (Cf) and Cyanine 5 (CY-5)) were coupled to the ε-amino group of Lys at the *C*-terminus, or directly on the N-terminus in solution using DIC and HOBt (Scheme 1). In the antitumor drug (methotrexate (MTX) or Succinylated daunomycin (DauSuc)) containing conjugates, the drugs were conjugated to the Lys side-chain in solution using DIC-HOBt coupling reagents (Scheme 1). 

The peptides and conjugates were characterized by ESI-MS and analytical RP-HPLC (Table 1; analytical RP-HPLC chromatograms and MS spectra are in the Supplementary Information, Figures S5–S44).

### 2.2. Cellular Uptake 

Cellular uptake of peptides was measured using flow cytometry on MCF-7 and MDA-MB-231 cells. Cells were treated with the fluorescently labelled peptides at 1, 5 and 10 µM concentrations for 90 min at 37 °C. The peptides showed concentration dependent uptake. 

**Figure 2:**
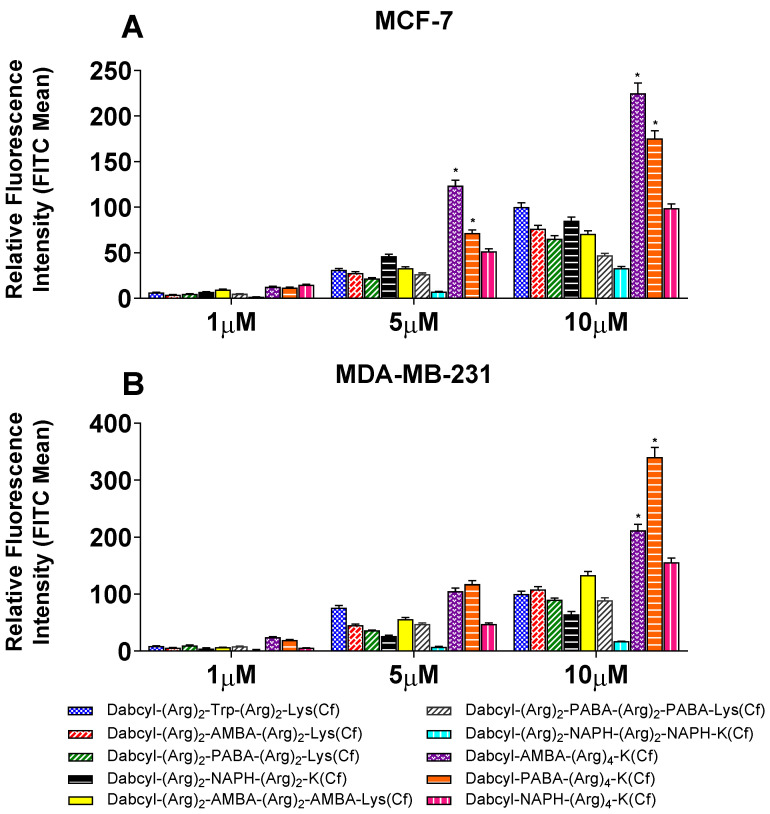
Cellular uptake of peptides on MCF-7 (**A**) and MDA-MB-231 (**B**) cells. Cells were treated with 1, 5 and 10 µM peptide solution for 90 min. The fluorescence intensity of the cells was determined by flow cytometry after trypsin treatment. The fluorescence intensity is relative to Dabcyl-RRWRRK(Cf) at 10 µM (100%). Differences between the peptides (Dabcyl-AMBA-RRRR-K(Cf) and Dabcyl-PABA-RRRRK(Cf)) and Dabcyl-RRWRRK(Cf) were determined by Student’s *t* test (* *p* < 0.05). Data represents the mean ± standard deviation (SD).

When AMBA or PABA was built into the tetraarginine sequence, the uptake was increased in the case of Dabcyl-AMBA-RRRRK(Cf) and Dabcyl-PABA-RRRRK(Cf) (Figure 2A). On MCF-7 cells, their internalization was at least twice as high as those of Dabcyl-RRWRRK(Cf) at 5 and 10 µM. On MDA-MB-231 cells, their uptake was significantly higher only at 10 µM (Figure 2B). However, it should be noticed that although Dabcyl-PABA-RRRRK was internalized in a similar manner to Dabcyl-AMBA-RRRRK on MCF-7, on MDA-MB-231 its uptake was exceptional (at 10 µM), as this peptide reached 340% relative uptake intensity. The other modifications of tetraarginine with AMBA or PABA in the middle or in both positions (*N*-terminus and middle) did not increase the internalization efficacy (Figure 2A,B).

Interestingly, the incorporation of NAPH into different positions did not show the same effect as the previous derivatives (Figure 2). The derivatives had similar or significantly decreased cellular uptake (in case only Dabcyl-RR-NAPH-RR-NAPH-K(Cf)). With resemblance to the previous conjugates, Dabcyl-NAPH-RRRRK(Cf) was able to induce the highest uptake among the NAPH modified peptides. Regardless, its uptake is overtaking Dabcyl-RRWRRK on MDA-MB-231 only at the highest concentration.

Dabcyl is well-known as a quencher in FRET pairs with Cf [37]. This feature may represent a challenge since during flow cytometry the detected fluorescence can be emitted mainly from cleaved peptides only, while non-cleaved peptides are not detected due to quenching. To assess the degree at which the fluorescence is depressed, we measured the fluorescence in the absence and the presence of trypsin (Supplementary Figure S1). This experiment has shown a high degree of quenching by Dabcyl.

**Figure 3:**
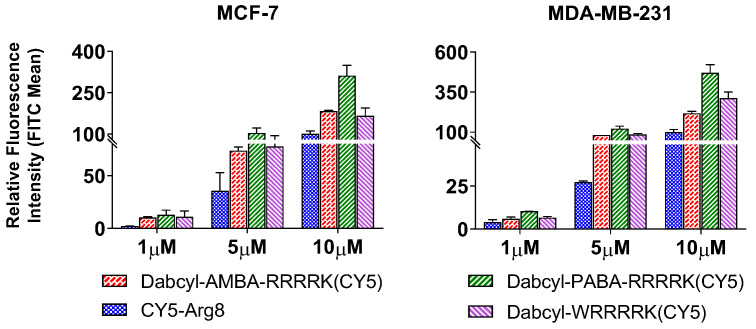
Cellular uptake of peptides on MCF-7 and MDA-MB-231 cells. Cells were treated with 1, 5 and 10 µM peptide solutions for 90 min. The fluorescence intensity of the cells was determined by flow cytometry after trypsin treatment. The fluorescence intensity is relative to Arg_8_ at 10 µM (100 %).

To refine our measurements, the best constructs (Dabcyl-AMBA-RRRRK and Dabcyl-PABA-RRRRK) were conjugated with cyanine 5 dye (CY-5), whose emission wavelength (662 nm) [38] is outside of the absorption of Dabcyl (343-567 nm). The cellular uptake of these labelled peptides was studied on MCF-7 and MDA-MB-231 cells (Figure 3). 

Both peptides were taken up to a great extent and they had better internalization than those of octaarginine, especially Dabcyl-PABA-RRRRK. This peptide showed remarkable uptake, mainly on MDA-MB-231 cells, as it was able to overtake the uptake of Arg8 at all concentrations, by several folds (3 to 4.5-folds). The AMBA derivative was also efficiently internalized, but it was taken up to a lesser extent PABA derivative. It overtakes the uptake of Arg8 by 2-folds at each concentration. 

**Figure 4:**
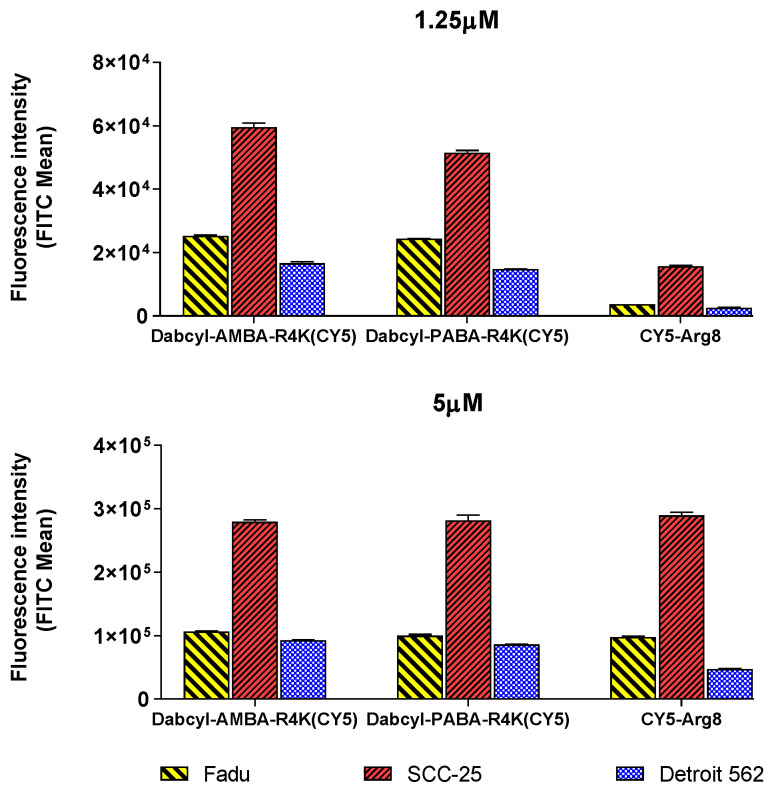
Cellular uptake of peptides on FADU, SCC-25, and Detroit 562 cells. Cells were treated with 1.25 and 5 µM peptide solutions for 60 min. The fluorescence intensity of the cells was determined by flow cytometry after trypsin treatment.

The uptake of these two efficient, Cy5-labelled peptides was studied further on other cell lines; FADU, SCC-25, and Detroit 562 (Figure 4). At low concentration both peptides had 2–3-fold higher cellular uptake than octaarginine on all cell lines. While at 5 µM they showed an equal uptake to Arg8 on Fadu and SCC-25 cells, their internalization was two-fold higher on Detroit 562 cells than that of octaarginine. An additional uptake study has also revealed that the constructs have time dependent cellular uptake (Supplementary Figure S2).

### 2.3. Cellular Distribution

**Figure 5:**
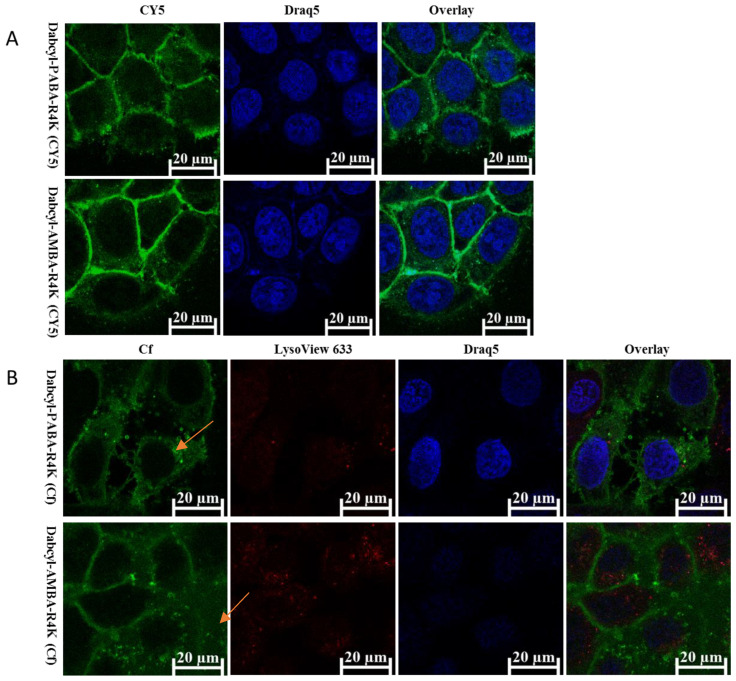
Cellular distribution of peptides on FADU cells visualized by confocal microscopy. Cells were incubated with the fluorescent peptide conjugates (5 µM). (**A**) Cells were treated with CY5-labelled peptides (green) for 1h and nuclei were stained with Draq5 (blue). (**B**) Visualization of vesicular uptake, cells were treated with Cf-labelled peptides (green) for 3 h and nuclei were stained with Draq5 (blue) while lysosomes were stained with LysoView 633. Imaging was performed by a Zeiss LSM 710 system. Orange arrows: unfamiliar bubble-like structures that formed after the treatment with the peptide.

**Figure 6:**
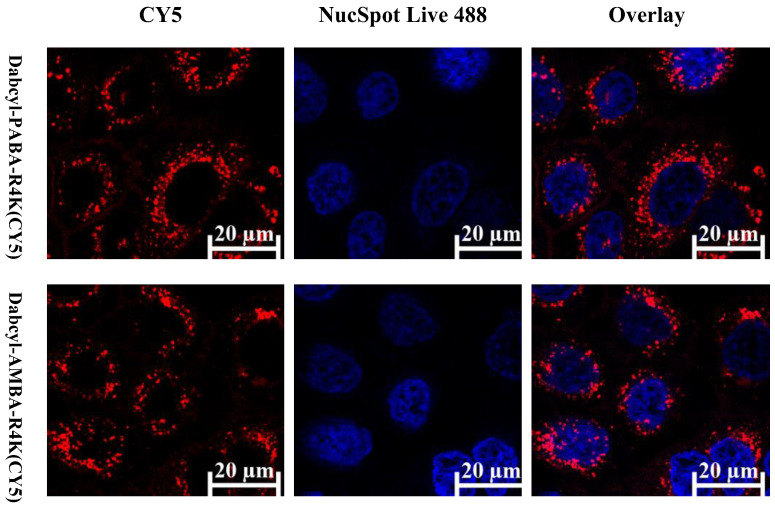
Cellular distribution of peptides on SCC-25 cells visualized by confocal microscopy. Cells were incubated with the CY5-labelled peptides for 1hr (Red) at 5 µM. Nuclei were stained with NucSpot Live 488 (blue). Imaging was performed by a Zeiss LSM 710 system.

**Figure 7:**
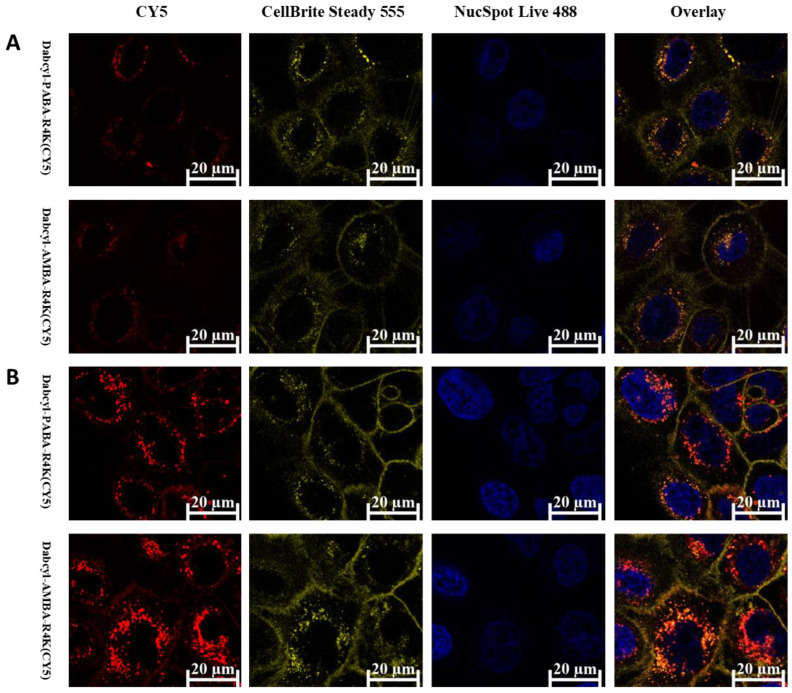

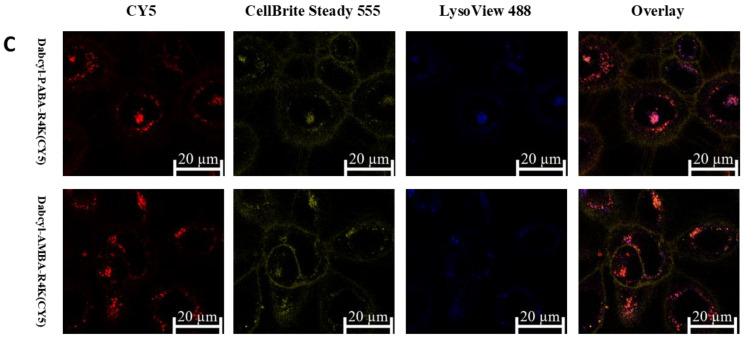
Cellular distribution of peptides on SCC-25 cells visualized by confocal microscopy. Cells were incubated with the CY5-labelled peptides for 1hr (Red). CellBrite Steady was used to stain the membrane (green). Nuclei were stained with NucSpot Live 488 (Figure 6A,B blue), lysosomes were stained with LysoView 488 (Figure 6C blue). Concentrations of the peptides were (**A**) 5 µM, (**B**) 0.31 µM and (**C**) 5 µM in the three experiments. Imaging was performed by a Zeiss LSM 710 system.

Confocal microscopy imaging was utilized to examine the uptake mechanisms and the distribution of peptide in cells. For this purpose, FADU and SCC-25 cells were treated with the fluorescent peptide conjugates at different times and concentrations (Figure 5, Figure 6 and Figure 7). On FADU cells, the CY5-labelled peptides showed moderately diffuse cytosolic uptake and strong membrane binding (Figure 5A). In an additional study FADU cells were treated with the Cf labelled peptides and a lysosomal dye to evaluate the lysosomal accumulation (Figure 5B). The majority of peptide was not colocalized with the lysosomal dye (Figure 5B). An odd finding was the unfamiliar bubble-like structures that formed after the treatment with the peptide (orange arrows in Figure 5B). 

The SCC-25 cells were treated at two concentrations with the cell-penetrating peptides (0.31 and 5 µM, Figure 6 and Figure 7), and in addition a membrane dye was included to assess the membrane binding of peptides (Figure 7B–D). The confocal study at 5 µM without membrane or lysosome dyes (Figure 6), was included to confirm that the presence of the organelle markers (membrane or lysosome) did not affect the internalization of the peptides.

Peptides were taken up at both concentrations although at higher concentration (5 µM) the uptake was much more intensive, showing concentration dependence. The peptide distribution was punctuated at both concentrations (Figure 6 and Figure 7A,B) and the uptake of both peptides was high. Both peptides showed vesicular uptake, as no diffuse fluorescence signal was detected. Moreover, the peptide did not reach the nucleus, but rather accumulated in its vicinity. 

To take this observation to a further step, SCC-25 cells were treated with an additional dye, to evaluate the lysosomal uptake of peptides (Figure 7C). The distribution of peptides and lysosomal dye reveals that the majority of the peptides are not colocalized with the lysosome. The degree of colocalization was higher in the case of Dabcyl-PABA-RRRRK peptide.

### 2.4. Investigation of Endocytic Pathways of Entry 

**Figure 8:**
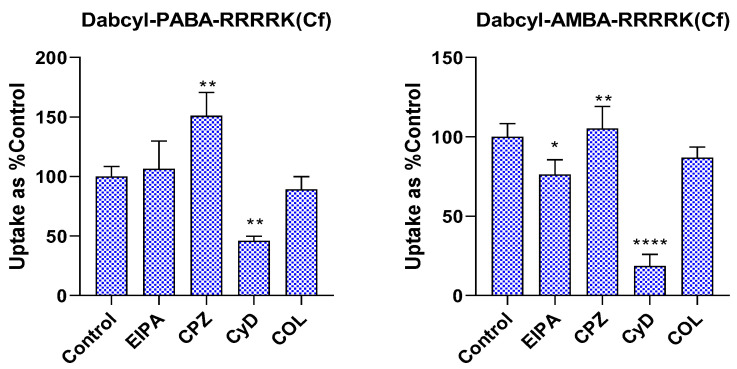
Effect of endocytosis inhibitors on the cellular uptake of peptide conjugates. MDA-MB-231 cells were pretreated with inhibitors EIPA (50 µM), CPZ (30 µM), CyD (5 mM) and COL (20 µM) for 30 min, the cells were then treated with the peptide conjugates (5 µM) for 90 min. Significant difference from control was measured using student’s *t* test (* *p* <0.05, ** *p* < 0.01and **** *p*< 0.0001).

Several endocytic routes can be used by CPPs for entry, which then determine their intracellular fate. The dependence of cellular uptake of the two most promising peptides, Dabcyl-PABA-RRRRK and Dabcyl-AMBA-RRRRK, on different inhibitors was examined on MDA-MB-231 cells (Figure 8). The following inhibitors were applied: 5-(N-ethyl-N-isopropyl)amiloride (EIPA) as micropinocytosis inhibitor [39], chlorpromazine (CPZ) to inhibit clathrin-mediated endocytosis [40], methyl-beta-cyclodextrin (CyD) to deplete cholesterol from the lipid rafts, and thus to inhibit caveolae-mediated endocytosis [41]. Finally, colchicine (COL) was used to ascertain if microtubules are involved in the uptake of the peptides [42] as this agent inhibits microtubule formation [43].

Treatment of peptides with EIPA showed a contrasting effect for peptides. While it decreased the uptake of Dabcyl-AMBA-RRRRK, it did not alter the internalization of Dabcyl-PABA-RRRRK. CPZ, a clathrin-mediated endocytosis inhibitor, and COL, a microtubule inhibitor, did not cause any inhibition. The most significant change was caused by CyD. It significantly reduced the uptake of Dabcyl-AMBA-RRRRK(Cf) and Dabcyl-PABA-RRRRK(Cf) to 18.7% and to 45.9% of the control (untreated), respectively. 

**Figure 9:**
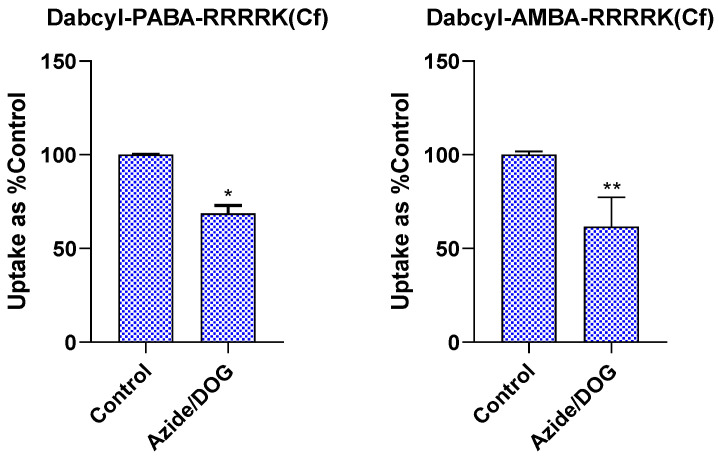
Effect of endocytosis inhibitors on the cellular uptake of peptide conjugates. MDA-MB-231 cells were pretreated with NaN_3_ (10 mM) and DOG (5 mM) for 30 min, the cells were then treated with the peptide conjugates (5 µM) for 90 min. Significant difference from control was measured using Student’s *t* test (* *p* < 0.05 and ** *p* < 0.01).

The energy independent pathways were also studied using sodium azide (NaN_3_) and 2-deoxyglucose (DOG) pretreatment, resulting in ATP depletion and thus the inhibition of energy dependent pathways (Figure 9).

Upon the pretreatment with NaN_3_ and DOG, the uptake was significantly lower. The internalization of Dabcyl-AMBA-RRRRK was reduced to a greater extent than Dabcyl-PABA-RRRRK. This experiment suggests the presence of a nonendocytic route for the peptides, as they are still taken up after the inhibition of endocytosis.

### 2.5. Fluorimetry Based Measurement of Cellular Uptake at 4 °C and 37 °C 

Another method to accurately measure the internalized peptide is the lysis of cells after accurate removal of extracellular and membrane-bound peptides, and subsequently measure the fluorescence intensity. Such a method can overcome bias related to the Dabcyl quenching of the fluorophore since the peptides are degraded by the cell-lysis released and added (trypsin) enzymes [32,33]. The internalization was quantified at 37 °C and 4 °C. At 4 °C the endocytic mechanisms of entry are inhibited [44]. Although at this low temperature, the membrane must be in the gel phase, early studies showed that cell penetrating peptides can translocate across the membrane at this low temperature as well [15]. Therefore, the uptake at 4 °C represents direct membrane translocation, while the difference in the uptake at 37 and 4 °C corresponds to endocytic mechanisms.

**Figure 10:**
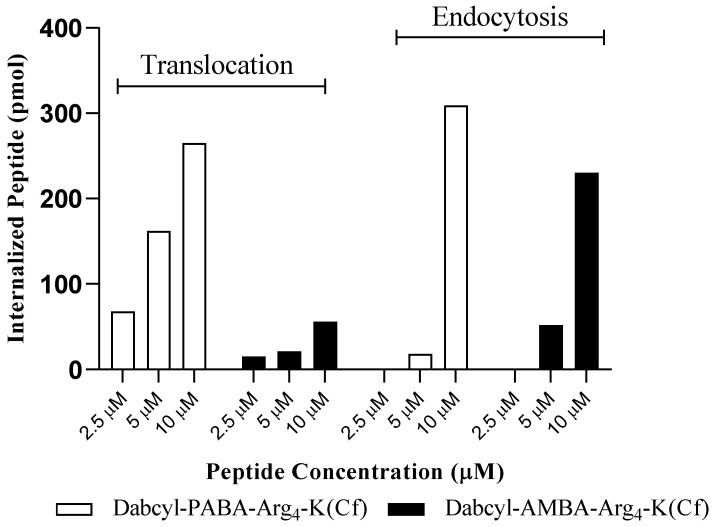
Cellular uptake of peptides into CHO cells by fluorometry. Cells at 37 °C and 4 °C were treated for 1 hr with concentrations 2.5, 5 and 10 µM. Cells were lysed with Tris 50mM, NP-40 1%, NaCl 1M and trypsin before fluorescence was measured (λ_ex_ = 494 nm and λ_em_ = 510–560 nm).

CHO cells were treated with Dabcyl-PABA-RRRRK and Dabcyl-AMBA-RRRRK peptides at 2.5, 5, and 10 µM concentration (Table 2 and Figure 10). Fluorescence signal quantification shows that Dabcyl-PABA-RRRRK peptide is superior in its uptake at all concentrations and is able to utilize translocation much more effectively than the other peptide.

To properly visualize the uptake, Figure 10 displays the uptake mode of the peptides, at each concentration. The PABA containing peptide, is not only more effective, but is able to internalize by translocation, at most concentrations, and almost exclusively by translocation at 5 µM. It seems that the peptide is taken up in a similar manner to the AMBA peptide in terms of endocytosis. However, the additional intense uptake comes from the large amount of peptide taken by translocation. The AMBA peptide is also capable of internalizing by translocation, where the internalized amount by translocation represents approximately one third of all internalized peptide at 5 µM. The ratio is much less at 10 µM for this peptide. 

### 2.6. Effect of Membrane Dipole Potential on the Cellular Uptake

**Figure 11:**
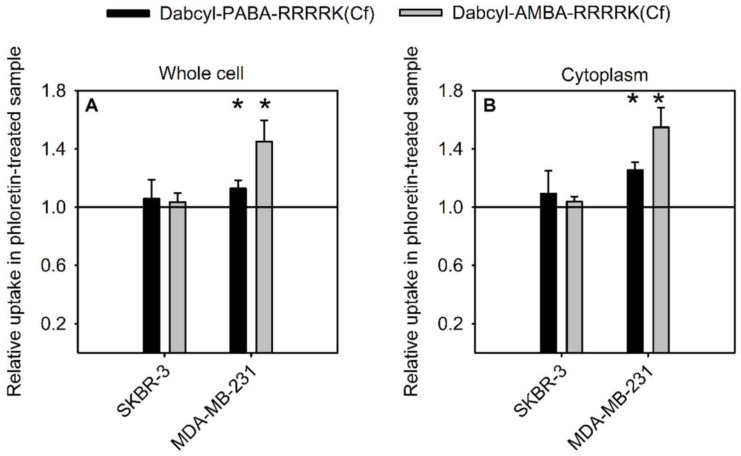
The uptake of peptides is influenced by the dipole potential. Cells were pretreated with 100 μM phloretin in the presence of 0.05% (*v/v*) Pluronic F-127 to decrease their dipole potential followed by incubating them with 5 μM peptide for 20 min. The fluorescence intensity of the peptides in the whole cell or in the cytoplasm was determined and normalized to the intensities of cells pretreated with Pluronic F-127 only. The results represent the mean (±SEM) of three independent biological replicates. Statistical significance was tested by two-way ANOVA followed by Sidak’s multiple comparison test, and the asterisks show a significant effect of phloretin treatment according to the posthoc test (* *p* < 0.05).

The cellular uptake of the two best constructs (Dabcyl-PABA-RRRRK(Cf) and Dabcyl-AMBA-RRRRK(Cf)) was examined in the presence of phloretin. This reagent decreases the positive dipole potential of the membrane [45] and it was shown to increase the uptake of penetratin [46]. Since both penetratin and the tetraarginine-based constructs are positively charged and consequently expected to be affected by the positive dipole potential, we studied the effect of phloretin on our peptides in two cell lines. While SKBR-3 cells were insensitive to phloretin, the uptake of both peptides was increased in MDA-MB-231 cells at the decreased dipole potential (Figure 11, Supplementary Figure S4). This effect was more pronounced if uptake into the cytosol was considered instead of total cellular uptake. 

### 2.7. In Vitro Cytostatic Effect of Conjugates

Since the peptides showed efficient internalization, their ability to deliver drug molecules was investigated. The construct with the arrangement that guarantees the highest uptake (Dabcyl-X-RRRRK) was conjugated with antitumor drugs (MTX and DauSuc). The drug molecules were attached to the ε-amino group of lysine at *C*-terminus. Pentaglutamylated MTX via a GFLG spacer was conjugated too (Dabcyl-AMBA-RRRRK(GFLG-E5-MTX)). These modifications were reported to increase the activity of CPP/MTX conjugates [6]. The in vitro cytostatic activity was measured on MCF-7 and MDA-MB-231 cells (Table 3). Based on the flow cytometry experiments, the peptides have no cytostatic effect on these cell lines.

In terms of DauSuc delivery, the peptides were able to inhibit the proliferation of cancer cells. The AMBA-containing peptide was clearly the most active peptide having the lowest IC_50_ values (10.3 µM on MCF-7 cells, and 10.1 µM on MDA-MB-231) and was more effective than the well-known peptide Arg_8_ and the Trp containing analogue. The other two peptides had the same or better activity as octaarginine and the Trp analogue respectively on MCF-7 cells. They were better than these two peptides on MDA-MB-231 cells.

While the MTX-Arg_8_ conjugate was ineffective, the MTX conjugate of all peptides were active as indicated by the IC_50_ values. Our constructs showed low IC_50_ values (as low as 8.0 µM on MCF-7 and 16.3 µM on MDA-MB-231). Moreover, the peptides seem to have higher activity on MCF-7 cells, unlike the Trp containing peptide, which is more active on MDA-MB-231 cell line. 

## 3. Discussion

The effect of amino acids with aromatic side-chains, mainly Trp was already shown to promote the uptake of oligoarginines [10,26]. In this work, we explored the influence of aromatic moieties in the backbone of the peptides on the cellular uptake. Our earlier results showed that Dabcyl-WRRRRK(Cf), Dabcyl-RRWRRK(Cf), and Dabcyl-RRWRRWK(Cf) peptides could be very efficiently internalized. It was shown that the positions of aromatic residues are essential in the uptake of Dabcyl-containing peptides [33]. Thus, aromatic residues were distributed in those CPPs using the same arrangements in the sequences. These aromatic residues can effectively influence the uptake to a higher degree than Trp.

The used unnatural amino acids (AMBA, PABA and NAPH), unlike Trp, insert aromaticity directly into the backbone. It turned out that using different arrangements affirmed our previous finding as the uptake is enhanced when the aromatic group is placed at the *N*-terminus (Figure 2). It is worthy to note that the rigidity due to the aromatic groups influences the cellular uptake. The presence of such groups results in planarity by electron delocalization, and thus structures with the aromatic groups in the core of the sequence may be more rigid. This may explain why placing such residues in the core of the sequence leads to poor uptake, and increasing the number of such residues (higher rigidity) can even decrease the uptake, and this is especially reflected in NAPH-containing peptides. Rigidity was shown to positively influence cellular uptake as Arg-rich peptides were shown to have enhanced uptake after increased rigidity with cyclisation [47]. However, this is not the case with constructs in this work. The resulting rigidity manifested as a consequence of inserting such groups in the backbone may not allow favorable conformations or interactions with the membrane. 

Since the peptides Dabcyl-AMBA-RRRRK and Dabcyl-PABA-RRRRK showed high uptake, we labelled such peptides with CY5, and compared their uptake with the well-known Arg8, on several cell lines. The peptides were noteworthy, as they exceeded the uptake of Arg8 by several folds (on MCF-7 and MDA-MB-231). On other cell lines (FADU, SCC-25, and Detroit 562), the peptides did not internalize as efficiently, yet they still overtook the uptake of Arg8, especially at low concentrations, highlighting the intense uptake of our constructs. It was already established that the presence of aromatic residues (especially Trp) favors high uptake in oligoarginines as a result of ion pair–π interactions [24]. It is logical to assume that the presence of PABA and AMBA groups promotes the same effect, enabling proper interaction with the membrane. Moreover, the proximity to Dabcyl is essential. The aromatic groups adjacent to Dabcyl may participate in the electronic interactions with the membrane as a single unit, in comparison to placing such residues in the middle of the sequence. 

Cellular distribution of peptides was different on FADU and SCC-25 cells. On FADU cells, the peptides show diffuse cellular uptake rather than vesicular (Figure 5A), and such peptides are not mainly captured by the lysosomes, since they do not colocalize with lysosomes (Figure 5B). This is contrasted with the uptake on SCC-25 cells. The peptides display mainly vesicular uptake at both concentrations (Figure 6 and Figure 7). However, though the uptake is vesicular on such cells, the imaging in the presence of a lysosomal marker suggests that the peptide is not located in the lysosomes. SCC-25 cells were reported to overexpress flotillin-1 [48], a protein residing in the lipid-rafts, which interacts with caveolin-1 [48]. Caveolin-1 is mainly involved in caveolae-mediated endocytosis [49]. We hypothesize that this may affect the vesicular uptake of the peptide on SCC-25 cells and have a higher tendency to take up the peptides via a lipid-raft-mediated process.

The preferred route of entry for Arg rich peptides was extensively studied. While some reported the main pathway to be clathrin-mediated endocytosis [50,51], others have reported it is micropinocytosis [52,53]. In our earliest work with Dabcyl group, our peptides (Dabcyl-containing oligoarginines) were shown to internalize by macropinocytosis [32]. The incorporation of Trp into the sequence has altered the endocytic route, we noticed the peptides tend to be internalized by caveolae/lipid raft-mediated endocytosis [33]. In this work, the replacement of Trp with AMBA or PABA did not influence the internalization mechanism, in fact Dabcyl-AMBA-RRRRK showed high dependence on this pathway (Figure 8). It is advantageous for the peptides to be taken up by caveolae/lipid raft-mediated endocytosis, as the vesicles formed in this route transfer their cargo to the Golgi apparatus and endoplasmic reticulum (ER), rather than lysosomes [54]. Thus, the peptides can reach the cell intact and avoid becoming entrapped by the lysosome. The presence of primary amphipathicity may have an influence on the behavior of the peptide, allowing the peptide to be taken up by this route. These results fit well with the confocal microscopy images since peptides with vesicular uptake did not colocalize with lysosomal markers (Figure 7C). This finding hints at the possibility that such peptides are taken up by non-lysosomal vesicles. For a cell-penetrating peptide to reach the cytosol, it must cross a membrane independent of whether it is taken up by endocytosis or direct membrane translocation. For positively charged cell-penetrating peptides, this step must be hindered by the dipole potential, which is positive inside the membrane [45]. Decreasing the dipole potential enhanced the cytosolic amount of the most effective peptides described in this paper in a cell-dependent way, confirming the role of the dipole potential established previously for penetratin [46].

Pretreatment with NaN_3_ and DOG has shown that our peptides can utilize energy independent pathways for internalization as well (Figure 9). Yet, the lysis experiment (Figure 10) has shown the significance of structure in cellular uptake. Up to 2.5 µM peptides generally internalize solely by direct penetration, but at higher concentrations, the majority of the peptide is taken up by endocytosis, as the case with the AMBA-containing peptide, and our earlier peptides [32,33]. For Dabcyl-PABA-RRRRK, it is almost exclusively taken up by translocation at 2.5 and 5 µM, and at 10 µM the peptide is taken up almost equally for each pathway. As we have measured the uptake for each pathway, it was evident that Dabcyl-PABA-RRRRK is a novel peptide since it relies on direct penetration, especially at 5µM concentration or lower. There are peptides which are able to internalize via translocation, but not to the degree that the PABA peptide is capable of, which relies mainly on translocation.

**Scheme 2:**
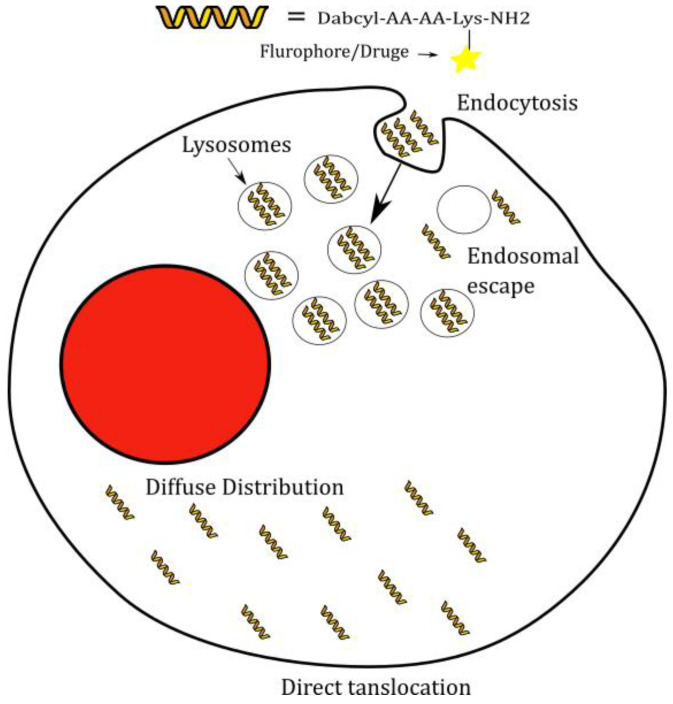
Schematic diagram of the main pathways of penetration.

The uptake pathways of such peptides are favorable. In terms of endocytosis, the vesicles formed by caveolae/lipid-rafts mediated endocytosis do not fuse with the lysosomes. In fact, this pathway is generally exploited by many pathogens, to avoid degradation in lysosomes [55]. On the other hand, the peptides were also able to exploit non-endocytic routes, as evidenced by the inhibition experiment and the lysis quantification experiment, which is significant, since the peptides can also avoid vesicular entrapment. In terms of uptake, our peptides can gain entry into the interior of cells, allowing them to be potential delivery vectors for different applications, such as drug delivery (Scheme 2). 

The prepared CPPs have displayed their ability to effectively deliver antitumor drugs to cancer cells, especially Dabcyl-AMBA-RRRRK (Table 3). This finding is intriguing as this peptide is less taken up than the PABA-containing peptide on such cell lines (MCF-7 and MDA-MB-231). This can be explained by the different properties of the peptides, which may need further studies. One interesting finding is the ability of such peptide/drug conjugates to affect MCF-7 cells. This is important since the Trp-containing conjugates were not effective on this cell line. Such a finding implies that our modifications (AMBA, PABA and NAPH) resulted in enhanced behavior and increased activity when compared to a natural residue.

## 4. Materials and Methods

### 4.1. Synthesis of Peptides and Their Conjugates

The peptides were synthesized by solid phase peptide synthesis (SPPS) on Rink amide MBHA resin, using the Fmoc/tBu strategy. The side-chain of amino acids was protected as follows: 2,2,4,6,7-pentamethyldihydrobenzofuran-5-sulfonyl (Pbf, in Arg), *tert*-butyl ester (O*^t^*Bu in Glu) and *tert*-butyloxycarbonyl (Boc) in Lys. The *N*-terminus Fmoc protecting group was cleaved by a cleavage solution of 2% piperidine and 2% 1,8-diazabicyclo [5.4.0]undec-7-ene (DBU) in dimethyl formamide (DMF) (2 + 2 + 5 + 10 min). Remains of the cleavage solution were washed out with DMF (8 × 1 min). For coupling, amino acid derivatives, DIC and HOBt as coupling reagents were used with 3 eq molar excess. The coupling reaction was carried out in DMF at RT for 60 min. The coupling of amino acids after the PABA or AMBA was performed using HATU (3eq) in presence of DIEA (3eq). After the coupling, the resin was washed with DMF (2 × 1 min) and dichloromethane (DCM) (3 × 1 min). To assure that the coupling proceeded with success, Kaiser test was performed and upon the confirmation of a successful reaction, the process is repeated until the desired sequence is completed. After the removal of the last Fmoc group Dabcyl or 5(6)-carboxyfluorescein Cf was attached to the peptide using HOBt/DIC coupling reagents in solution. The cleavage of Mtt from lysine side chain was achieved by using 2% TFA in DCM (5 + 5 + 10 + 30 min). The peptide resin was then washed with DIEA in DCM (3 × 5 min) to neutralize it for the next coupling, followed by washing with DMF (8 × 1 min), and then by a coupling step. The peptides were cleaved from the resin with 5 mL TFA containing 0.365 g phenol, 0.25 mL distilled water, 0.25 mL thioanisole and 0.125 mL EDT as scavengers. Crude products were precipitated by dry diethyl-ether, dissolved in 10% acetic acid, lyophilized, and further purified by semi preparative HPLC. The purified compounds were characterized by analytical RP-HPLC and ESI-MS. The purity of the compounds was higher than 95%.

Lys residue was included in peptides to attach the Cf, methotrexate (MTX) or succinylated daunomycin (DauSuc). Peptide conjugates of Cf, MTX, or DauSuc were synthesized by reacting the peptide with 1 eq of dye or drug, 1.1 eq of DIC and HOBT as coupling reagents in the presence of 2 eq of *N,N*-diisopropylethylamine (DIEA), in DMF and the reaction was stirred overnight. Eluent A was added to the reaction mixture in appropriate amount and purified by RP-HPLC.

To couple the CY5 dye to peptides, both the N-hydroxysuccinimide ester of dye and the peptide were dissolved in DMF in a 1:1 ratio. Then, 3 eq of DIEA as a base was added to the solution. The reaction mixture was stirred overnight and purified by RP-HPLC after the evaporation of DMF.

### 4.2. Flow Cytometry

#### 4.2.1. MCF-7 and MDA-MB-231

To study the cellular uptake of fluorophore-labelled peptides, 10^5^ cells per well were plated on 24-well plates. Cells were incubated for 24 h at 37 °C, followed by treatment with the peptides at different concentrations, in serum-free medium for different time intervals. For negative control, cells were treated with serum-free medium. After the incubation, the peptide solutions were removed and 100 µL trypsin (0.25%) was added for 5–10 min to remove membrane bound peptides and detach the adherent cells from the plates. The activity of trypsin was terminated by the addition of 900 µL HPMI buffer (glucose, NaHCO_3_, NaCl, HEPES, KCl, MgCl_2_, CaCl_2_, Na_2_HPO_4_·2H_2_O) containing 10% fetal bovine serum, and the cells were transferred to tubes for measurement. The cells were centrifuged at 216 g at 4 °C and the supernatant was disposed. Cells were re-suspended in 300 µL HPMI. The fluorescence intensity of cells was quantified by flow cytometer (BD LSR II, BD Bioscience, San Jose, CA, USA). Data were analyzed with FACSDiVa 5.0 software.

The effect of inhibitors was studied in the same manner but with pretreatment by an inhibitor for 30 min, and subsequently treated with a peptide conjugate at 5 µM. The cells were then incubated for 90 min at 37 °C. Macropinocytosis was inhibited using 5-(*N*-Ethyl-*N*-isopropyl)amiloride (EIPA) [39], clathrin-mediated endocytosis with chlorpromazine (CPZ) [40], colchicine (Col) to assess the role of microtubules [42], and methyl-β-cyclodextrin (CyD) for the inhibition of caveolae/lipid-raft-mediated endocytosis [41].

In order to inhibit all pathways of endocytosis, MDA-MB-231 cells were pretreated with sodium azide (NaN_3_) (10 mM) and 2-Deoxyglucose (DOG) (5 mM) for 30 min and the peptide was added to the wells. The cells were then incubated for 90 min at 37 °C.

#### 4.2.2. Detroit 562, FaDu and SCC-25

Briefly, 4 × 10^4^ cells per well were seeded onto 24-well plates (Corning Costar cell culture plate, TC treated, flat bottom) and allowed to adhere for 48 h. Cells were treated with the fluorophore-labelled peptides at the desired concentrations in the appropriate serum free medium, and incubated in a humidified, 5% CO_2_ atmosphere incubator for the specified period at 37 °C. After incubation, supernatant was discarded, and the cells were washed twice with PBS. Trypsin was added for 10 min at 37 °C. Cells were pelleted (150× *g*, 5 min, 4 °C) in flow cytometry tubes, resuspended in 300 μL PBS, and kept at 4 °C for the rest of the experiment. The fluorescence intensity of the conjugate-stained cells was analyzed using a CytoFLEX flow cytometer (Beckman Coulter, Brea, CA, USA) with 638-nm laser and APC-A 660/10 BP filter. Data were evaluated using CytExpert software (version 2.3.0.84).

### 4.3. Internalization Quantification by Fluorometry of Lysed Cells at 37 °C or 4 °C

We used the quantification method described previously [32,33]. Briefly, one million CHO cells were incubated for 1 h at 37 °C (or 4 °C) with the fluorescent peptides in 500 µL DMEM-F12. After incubation with peptides and washing cells with HBSS, 500 µL 0.05% trypsin/EDTA 0.05% (37 °C) or 500 µL pronase 0.05% in 100 mM Tris pH 7.4 (4 °C) was added for 5 min to hydrolyze the remaining extracellular peptide, the membrane-bound peptide and to detach cells. After the addition of 100 µL protease inhibitor (complete mini at 4 °C (Roche) or trypsin inhibitor (soybean inhibitor 5 mg/mL) at 37 °C mixed with 100 µL bovine serum albumin (1 mg/mL), cells were transferred into a microtube, centrifuged, washed with 1 mL 50 mM Tris buffer pH 7.4, 0.1% BSA, and lysed in 200 µL 50 mM Tris pH 7.4, 1 M NaCl, 1% Nonidet P40 plus trypsin to cleave the peptide and recover the whole associated fluorescence signal. The samples were then sonicated for 15 min (to homogenize samples) and centrifugated for 10 min at 16,000× *g*. Fluorescence intensity in the supernatants was monitored with a MOS 200M fluorimeter (Biologic, Seyssinet-Pariset, France) (excitation at 494 nm and emission recorded between 510 and 560 nm). The maximal intensity around 525 nm was retained for the calibration curve and for quantification of samples. The quantities of internalized peptides were calculated by the fluorescence intensity of the sample with the calibration curve, for which we prepared a range of peptide amounts (from 2 to 500 pmoles) in the lysis buffer (50 mM Tris pH 7.4, 1 M NaCl, 1% Nonidet P40, trypsin) in the presence of one million suspended cells. The samples were sonicated for 15 min and centrifuged at 16,000× *g* for 10 min.

### 4.4. Confocal Microscopy

Fadu and SCC-25 cells were seeded into eight well Ibidi^®^ μ-Slide microscopic slides (Ibidi GmbH, Gräfelfing, Germany) and cultured as described in the Supplementary. After 48 h incubation, medium was removed, and cells were treated with the fluorophore-labelled peptides at the desired concentrations in the appropriate serum free medium. In the case of Cf-labelled peptides, DRAQ5 (ThermoFisher, Waltham, MA, USA) and Lysoview633 (Biotium, Fremont, CA, USA) fluorescent probes were applied. For nuclear counterstaining DRAQ5 was used in 5 µM final concentration. For lysosomal counterstaining Lysoview633 was used in 1:1000 dilution. Fluorescent probes were diluted in Cf-labelled peptide-containing medium and added to the cells 1 h before the end of the peptide treatment. In the case of Cy5-labelled peptides, NucSpot Live 488 (Biotium, Fremont, CA, USA), CellBrite Steady 550 (Biotium, Fremont, CA, USA), and LysoView 488 (Biotium, Fremont, CA, USA) fluorescent probes were applied. For nuclear counterstaining, NucSpot 488, for membrane counterstaining, CellBrite Steady 550, and for lysosomal counterstaining, LysoView 488 were used. All three probes were diluted in 1:1000 ratio in the Cy5-labelled peptide-containing medium and added to the cells 1 h before the end of the peptide treatment. After the treatments, cells were washed with PBS twice. Finally, PBS was added. Images were acquired with Zeiss Confocal LSM 710 microscope (Carl Zeiss AG, Oberkochen, Germany). Objective: Plan-Apochromat 63×/1.40 Oil DIC M27. Pinhole: 1.01 AU. Cf was excited at λ = 488 nm, and its emission was detected at λ = 490–533 nm, while the fluorescence of LysoView633 was excited at λ = 633 nm and monitored at λ = 661–730 nm, finally the DRAQ5 was exacted at λ = 543 mn and its emission was detected at λ = 593–622. Images were processed by software Zeiss ZEN Lite (version 2.3).

### 4.5. Study of the Effect of Membrane Dipole Potential on the Cellular Uptake

MDA-MB231 and SKBR-3 cells, seeded in Ibidi^®^ μ-Slide chambered coverslips (Ibidi GmbH, Gräfelfing, Germany), were treated with 100 μM phloretin (Sigma-Aldrich, catalog number: P7912, Merck KGaA, Darmstadt, Germany) in the presence of 0.05% (*v/v*) Pluronic F127 (Sigma-Aldrich, catalog number: P2443, Merck KGaA, Darmstadt, Germany) for 60 min to reduce their dipole potential. These treated cells and their untreated counterparts were incubated with the cell penetrating peptides at a concentration of 5 μM for 20 min followed by their confocal microscopic investigation using a ZEISS LSM880 microscope. The fluorescence of the Dabcyl fluorophore was excited at 488 nm and measured in the range between 490–562 nm. Images were analyzed in MATLAB to determine the cytoplasmic and total cellular uptake. A convolutional neural network was trained to recognize cells based on the transmission images. Fluorescence within this mask was determined and it corresponds to the total cellular fluorescence. Edge pixels were removed from these masks followed by removing pixels surrounded by a neighborhood of high variance of the fluorescence intensity (endosomes). This final mask corresponds to the cytoplasm. The image analysis procedure is demonstrated in Supplementary Figure S3.

### 4.6. Analysis of In Vitro Cytostatic Activity of Conjugates

Cells were grown to confluency and were plated into 96-well plates with initial cell number of 5 × 10^3^ per well. The cells were incubated at 37 °C for 24 h. Then, cells were treated with the compounds at 0.4–100 µM concentration range for 3 h in 200 µL as the final volume. Control cells were treated with serum free medium at 37 °C for 3 h. After the incubation, the cells were washed with serum free medium twice. To analyze the in vitro cytostatic effect, cells were further cultured for 72 h in serum containing medium. Then, the MTT assay was performed to measure the IC_50_ values. Hence, 45 µL of MTT solution was added to each well (2 mg/mL, serum-free medium used as a solvent). The cells were incubated for 4 h. Then, the plates were centrifuged at 900× *g* for 5 min, and the supernatant was discarded. To dissolve the formed purple crystals, 100 µL of DMSO was added, and the absorbance was determined at λ = 540 nm and 620 nm using ELISA plate reader (iEMS Reader, Labsystems, Finland). To measure the percentage of cytostasis, the following equation was used for calculation: Cytostatic effect (%) = [1 − (OD_treated_/OD_control_)] × 100; where OD_treated_ and OD_control_ correspond to the optical densities of the treated and untreated cells, respectively. In each case, three independent experiments were carried out with 4 parallel measurements. The 50% inhibitory concentration (IC_50_) values were determined from the dose-response curves. The curves were defined using MicrocalTM Origin (version 9.2) software: cytostasis was plotted as a function of concentration, fitted to a sigmoidal curve, and based on this curve, the IC_50_ value was determined. IC_50_ represents the concentration of a compound required for 50% inhibition in vitro and expressed as micromolar units.

## 5. Conclusions

This work illustrates the synthesis of efficient, short CPPs. The Dabcyl-containing oligoarginines were rendered effective by our modifications. The modifications are based on the insertion of aromatic, unnatural residues AMBA, PABA, and NAPH. By combining Dabcyl and one of those molecules, we were able to prepare peptides that can exceed the uptake of Arg8, with only four Arg residues, even though it was considered that oligoarginines with less than six Arg residues have low cellular uptake [18]. Such modifications enable activity at least as high as the natural residue Trp. Investigations into the mechanisms of internalization have revealed that such peptides internalize via favorable pathways which do not lead to lysosomal degradation. The peptides were even shown to have selective behavior according to the treated cell line (e.g., vesicular uptake in SCC-25 cells, and diffuse uptake in FADU cells). This may allow the peptides to be used for different applications according to the cell line. Finally, the peptides have demonstrated their ability to deliver antitumor drugs and an impact on both cell lines (MCF-7 and MDA-MB-231), unlike the Trp containing peptides, which were only effective against one cell line. In essence, our modifications produced effective CPPs that can be considered for further applications. The important finding in this work is the discovery of a peptide that can prefer direct penetration over endocytosis, resulting in changes in activity influenced by structure.

## Figures and Tables

**Table 1:** Chemical characterization of peptide conjugates.

Sequence	R_t_	M_calc_	M_meas_
Cf-Arg_8_^a^	11.4	1623.9	1624.4
Dabcyl-(Arg)_2_-Trp-(Arg)_2_-Lys(Cf)	14.7	1564.8	1565.6
Dabcyl-AMBA-(Arg)_4_-Lys(Cf)	14.9	1511.7	1511.6
Dabcyl-(Arg)_2_-AMBA-(Arg)_2_-Lys(Cf)	13.7	1511.7	1512.0
Dabcyl-(Arg)_2_-AMBA-(Arg)_2_-AMBA-Lys(Cf)	13.9	1644.8	1644.8
Dabcyl-NAPH-(Arg)_4_-Lys(Cf)	16.2	1547.7	1547.7
Dabcyl-(Arg)_2_-NAPH-(Arg)_2_-Lys(Cf)	13.8	1547.7	1547.7
Dabcyl-(Arg)_2_-NAPH-(Arg)_2_-NAPH-Lys(Cf)	14.2	1716.8	1716.4
Dabcyl-(Arg)_2_-PABA-(Arg)_2_R-Lys(Cf)	13.6	1497.7	1497.6
Dabcyl-PABA-(Arg)_4_-Lys(Cf)	15.3	1497.7	1497.3
Dabcyl-(Arg)_2_-PABA-(Arg)_2_-PABA-Lys(Cf)	13.6	1616.8	1616.8
CY5-Arg_8_^b^	15.2	1731.1	1731.4
Dabcyl-PABA-(Arg)_4_-Lys(CY5)^b^	17.7	1605.0	1605.2
Dabcyl-AMBA-(Arg)_4_-Lys(CY5)^b^	17.6	1619.0	1619.1
Dabcyl-AMBA-(Arg)_4_-Lys(DauSuc)	16.1	1761.9	1761.6
Dabcyl-PABA-(Arg)_4_- Lys(DauSuc)	16.5	1747.8	1747.5
Dabcyl-NAPH-(Arg)_4_-Lys(DauSuc)	16.9	1797.9	1798.1
Dabcyl-AMBA-(Arg)_4_-Lys(MTX)	13.6	1589.8	1589.6
Dabcyl-PABA-(Arg)_4_-Lys(MTX)	13.9	1575.8	1575.6
Dabcyl-NAPH-(Arg)_4_-Lys(MTX)	14.6	1625.8	1625.6
Dabcyl-AMBA-(Arg)_4_-Lys(GFLG-E5-MTX)	14.1	2609.3	2608.4

Analytical RP–HPLC was performed on Hypersil Hypurity C18 column (4.6 mm × 150 mm, 5 μm, 190 Å). The applied linear gradient elution was 0 min 0% B, 2 min 0% B, 22 min 90% B at 1 mL/min flow rate. The detection was carried out at λ = 220 nm. ^a^ analytical RP–HPLC was performed on YMC ODS-A C18 column (4.6 mm × 150 mm, 3 μm, 100 Å). The applied linear gradient elution was 0 min 0% B, 2 min 0% B, 22 min 90% B at 1 mL/min flow rate. The detection was carried on at λ = 220 nm. The mass spectrometric analysis was performed on a Bruker Daltonics Esquire 3000 plus (Germany). The samples were dissolved in acetonitrile–water (50:50, *v/v*), containing 0.1% acetic acid. ^b^ The mass spectrometric analysis was performed on a Bruker Amazon SL (Bremen, Germany). The samples were dissolved in acetonitrile–water (50:50, *v/v*), containing 0.1% acetic acid.

**Table 2:** Quantification of the extent of different internalization pathways in CHO cells (at 2.5, 5 and 10 µM). Values refer to the average amount of internalized peptide in pmoles. For each condition, experiments have been repeated independently at least 3 times.

Peptide	Conc.	Total Uptake (37 °C)	Direct Translocation (4 °C)	Endocytosis (Difference between Uptake at 37 and 4 °C)
Dabcyl-PABA-Arg_4_-K(Cf)	2.5 µM	50 ± 9	68 ± 14	0
	5 µM	180 ± 24	162 ± 33	18
	10 µM	574 ± 100	265 ± 70	309
Dabcyl-AMBA-Arg_4_-K(Cf)	2.5 µM	15 ± 5	15 ± 5	0
	5 µM	73 ± 9	21 ± 5	52
	10 µM	286 ± 19	56 ± 14	230

**Table 3:** Cytostatic activity of peptide/drug conjugates on MCF-7 and MDA-MB-231.

Conjugate	IC_50_ ±SD (µM) ^a^	
	MCF-7	MDA-MB-231
Dabcyl-AMBA-Arg-Arg-Arg-Arg-Lys(DauSuc)	10.3 ± 5.0	10.1 ± 6.0
Dabcyl-PABA- Arg-Arg-Arg-Arg-Lys(DauSuc)	17.7 ± 1.2	14.3 ± 1.1
Dabcyl-NAPH- Arg-Arg-Arg-Arg-Lys(DauSuc)	15.0 ± 4.2	16.6 ± 0.5
Dabcyl-Trp-Arg-Arg-Arg-Arg-Lys(DauSuc) ^b^	26.4 ± 4.2	18.0 ± 6.7
DauSuc-Arg_8_^b^	14.4 ± 0.2	19.5 ± 0.4
Dabcyl-AMBA-Arg-Arg-Arg-Arg-Lys(MTX)	11.8 ± 6.9	19.2 ± 7.1
Dabcyl-PABA- Arg-Arg-Arg-Arg-Lys(MTX)	18.6 ± 3.3	16.8 ± 4.7
Dabcyl-NAPH- Arg-Arg-Arg-Arg-Lys(MTX)	14.8 ± 2.5	16.9 ± 2.8
Dabcyl-AMBA-Arg-Arg-Arg-Arg-Lys(GFLG-E5-MTX)	8.0 ± 2.4	16.3 ±0.7
Dabcyl-Trp-Arg-Arg-Arg-Arg-Lys(MTX) ^b^	36.9 ± 3.0	7.4 ± 2.2
MTX-Arg_8_^c^	>100	>100
DauSuc ^d^	24.5 ± 7.1	>100
MTX ^c^	0.8 ± 0.3	>100

^a^ The cells were incubated with the compound for 3 h, and subsequently cultured in serum containing medium for 3 days.The IC_50_ values were determined by MTT assay as described in the text. Standard deviation values (SD) are also presented. ^b^ From the literature [33], ^c^ From the literature [6] and ^d^ From the literature [32].

## Data Availability

Not applicable.

## Supplementary Materials

The following supporting information can be downloaded at: https://www.mdpi.com/article/10.3390/pharmaceutics15010141/s1, Figure S1: The effect of cleavage of peptides by trypsin on fluorescence intensity.; Figure S2: Time dependence of peptides on FADU, SCC-25, and Detroit 562 cells.; Figure S3: Image segmentation for identification of cells and the cytoplasm.; Figure S4: Representative microscopic images showing the uptake of peptides and the effect of the diminished dipole potential.; Figure S5–S23: HPLC chromatogram of compounds. Figure S24–S44: MS Spectrum of compounds. References [56,57] are cited in Supplementary Materials.

## References

[1] Bánóczi Z., Gorka-Kereskényi Á., Reményi J., Orbán E., Hazai L., Tokési N., Oláh J., Ovádi J., Béni Z., Háda V. (2010). Synthesis and in vitro antitumor effect of vinblastine derivative- oligoarginine conjugates. Bioconjug. Chem..

[2] Miklán Z., Orbán E., Csík G., Schlosser G., Magyar A., Hudecz F. (2009). New daunomycin-oligoarginine conjugates: Synthesis, characterization, and effect on human leukemia and human hepatoma cells. Biopolymers.

[3] Bánóczi Z., Keglevich A., Szabó I., Ranđelović I., Hegedüs Z., Regenbach F.L., Keglevich P., Lengyel Z., Gorka-Kereskényi Á., Dubrovay Z. (2018). The effect of conjugation on antitumor activity of vindoline derivatives with octaarginine, a cell-penetrating peptide. J. Pept. Sci..

[4] Miklán Z., Szabó R., Zsoldos-Mády V., Reményi J., Bánóczi Z., Hudecz F. (2007). New ferrocene containing peptide conjugates: Synthesis and effect on human leukemia (HL-60) cells. Biopolym.—Pept. Sci. Sect..

[5] Miklán Z., Orbán E., Bánóczi Z., Hudecz F. (2011). New pemetrexed-peptide conjugates: Synthesis, characterization and in vitro cytostatic effect on non-small cell lung carcinoma (NCI-H358) and human leukemia (HL-60) cells. J. Pept. Sci..

[6] Szabó I., Orbán E., Schlosser G., Hudecz F., Bánóczi Z. (2016). Cell-penetrating conjugates of pentaglutamylated methotrexate as potential anticancer drugs against resistant tumor cells. Eur. J. Med. Chem..

[7] Bánóczi Z., Alexa A., Farkas A., Friedrich P., Hudecz F. (2008). Novel cell-penetrating calpain substrate. Bioconjug. Chem..

[8] Bánoczi Z., Tantos Á., Farkas A., Tompa P., Friedrich P., Hudecz F. (2007). Synthesis of cell-penetrating conjugates of calpain activator peptides. Bioconjug. Chem..

[9] Futaki S., Arafiles J.V.V., Hirose H. (2020). Peptide-assisted intracellular delivery of biomacromolecules. Chem. Lett..

[10] Jobin M.-L., Blanchet M., Henry S., Chaignepain S., Manigand C., Castano S., Lecomte S., Burlina F., Sagan S., Alves I.D. (2015). The role of tryptophans on the cellular uptake and membrane interaction of arginine-rich cell penetrating peptides. Biochim. Biophys. Acta.

[11] Jones S.W., Christison R., Bundell K., Voyce C.J., Brockbank S.M.V., Newham P., Lindsay M.A. (2005). Characterisation of cell-penetrating peptide-mediated peptide delivery. Br. J. Pharmacol..

[12] Hudecz F., Bánóczi Z., Csík G. (2005). Medium-sized peptides as built in carriers for biologically active compounds. Med. Res. Rev..

[13] Frankel A.D., Pabo C.O. (1988). Cellular uptake of the tat protein from human immunodeficiency virus. Cell.

[14] Vivès E., Brodin P., Lebleu B. (1997). A truncated HIV-1 Tat protein basic domain rapidly translocates through the plasma membrane and accumulates in the cell nucleus. J. Biol. Chem..

[15] Derossi D., Joliot A.H., Chassaing G., Prochiantz A. (1994). The third helix of the Antennapedia homeodomain translocates through biological membranes. J. Biol. Chem..

[16] Ziegler A., Nervi P., Dürrenberger M., Seelig J. (2005). The cationic cell-penetrating peptide CPPTAT derived from the HIV-1 protein TAT is rapidly transported into living fibroblasts: Optical, biophysical, and metabolic evidence. Biochemistry.

[17] Schmidt N., Mishra A., Lai G.H., Wong G.C.L. (2010). Arginine-rich cell-penetrating peptides. FEBS Lett..

[18] Khalil I.A., Kogure K., Futaki S., Harashima H. (2006). High density of octaarginine stimulates macropinocytosis leading to efficient intracellular trafficking for gene expression. J. Biol. Chem..

[19] Szabó I., Yousef M., Soltész D., Bató C., Mező G., Bánóczi Z. (2022). Redesigning of Cell-Penetrating Peptides to Improve Their Efficacy as a Drug Delivery System. Pharmaceutics.

[20] Caesar C.E.B., Esbjörner E.K., Lincoln P., Nordén B. (2006). Membrane interactions of cell-penetrating peptides probed by tryptophan fluorescence and dichroism techniques: Correlations of structure to cellular uptake. Biochemistry.

[21] Walrant A., Correia I., Jiao C.Y., Lequin O., Bent E.H., Goasdoué N., Lacombe C., Chassaing G., Sagan S., Alves I.D. (2011). Different membrane behaviour and cellular uptake of three basic arginine-rich peptides. Biochim. Biophys. Acta-Biomembr..

[22] Bechara C., Pallerla M., Zaltsman Y., Burlina F., Alves I.D., Lequin O., Sagan S. (2013). Tryptophan within basic peptide sequences triggers glycosaminoglycan- dependent endocytosis. FASEB J..

[23] Bechara C., Pallerla M., Burlina F., Illien F., Cribier S., Sagan S. (2015). Massive glycosaminoglycan-dependent entry of Trp-containing cell-penetrating peptides induced by exogenous sphingomyelinase or cholesterol depletion. Cell. Mol. Life Sci..

[24] Walrant A., Bauzá A., Girardet C., Alves I.D., Lecomte S., Illien F., Cardon S., Chaianantakul N., Pallerla M., Burlina F. (2020). Ionpair-π interactions favor cell penetration of arginine/tryptophan-rich cell-penetrating peptides. Biochim. Biophys. Acta-Biomembr..

[25] Shirani A., Mojarrad J.S., Farkhani S.M., Khosroshahi A.Y., Zakeri-Milani P., Samadi N., Sharifi S., Mohammadi S., Valizadeh H. (2015). The relation between thermodynamic and structural properties and cellular uptake of peptides containing tryptophan and arginine. Adv. Pharm. Bull..

[26] Rydberg H.A., Matson M., Amand H.L., Esbjörner E.K., Nordén B. (2012). Effects of tryptophan content and backbone spacing on the uptake efficiency of cell-penetrating peptides. Biochemistry.

[27] Zakeri-Milani P., Farkhani S.M., Shirani A., Mohammadi S., Mojarrad J.S., Akbari J., Valizadeh H. (2017). Cellular uptake and anti-tumor activity of gemcitabine conjugated with new amphiphilic cell penetrating peptides. EXCLI J..

[28] Khemaissa S., Walrant A., Sagan S. (2022). Tryptophan, more than just an interfacial amino acid in the membrane activity of cationic cell-penetrating and antimicrobial peptides. Q. Rev. Biophys..

[29] Tyagi S., Bratu D.P., Kramer F.R. (1998). Multicolor molecular beacons for allele discrimination. Nat. Biotechnol..

[30] Moss M.L., Miller M.A., Vujanovic N., Yoneyama T., Rasmussen F.H. (2016). Fluorescent substrates for ADAM15 useful for assaying and high throughput screening. Anal. Biochem..

[31] Tsuji M., Ueda S., Hirayama T., Okuda K., Sakaguchi Y., Isono A., Nagasawa H. (2013). FRET-based imaging of transbilayer movement of pepducin in living cells by novel intracellular bioreductively activatable fluorescent probes. Org. Biomol. Chem..

[32] Szabó I., Illien F., Dókus L.E., Yousef M., Baranyai Z., Bősze S., Ise S., Kawano K., Sagan S., Futaki S. (2021). Influence of the Dabcyl group on the cellular uptake of cationic peptides: Short oligoarginines as efficient cell-penetrating peptides. Amino Acids.

[33] Yousef M., Szabó I., Biri-Kovács B., Szeder B., Illien F., Sagan S., Bánóczi Z. (2021). Modification of Short Non-Permeable Peptides to Increase Cellular Uptake and Cytostatic Activity of Their Conjugates. ChemistrySelect.

[34] Futaki S., Suzuki T., Ohashi W., Yagami T., Tanaka S., Ueda K., Sugiura Y. (2001). Arginine-rich peptides. An abundant source of membrane-permeable peptides having potential as carriers for intracellular protein delivery. J. Biol. Chem..

[35] Letoha T., Gaá S., Somlai C., Czajlik A., Perczel A., Penke B. (2003). Membrane translocation of penetratin and its derivatives in different cell lines. J. Mol. Recognit..

[36] Delaroche D., Aussedat B., Aubry S., Chassaing G., Burlina F., Clodic G., Bolbach G., Lavielle S., Sagan S. (2007). Tracking a new cell-penetrating (W/R) nonapeptide, through an enzyme-stable mass spectrometry reporter tag. Anal. Chem..

[37] Alvarado-González M., Gallo M., Lopez-Albarran P., Flores-Holguín N., Glossman-Mitnik D. (2012). DFT study of the interaction between the conjugated fluorescein and dabcyl system, using fluorescene quenching method. J. Mol. Model..

[38] Lansdorp P.M., Smith C., Safford M., Terstappen L.W.M.M., Thomas T.E. (1991). Single laser three color immunofluorescence staining procedures based on energy transfer between phycoerythrin and cyanine 5. Cytometry.

[39] Koivusalo M., Welch C., Hayashi H., Scott C.C., Kim M., Alexander T., Touret N., Hahn K.M., Grinstein S. (2010). Amiloride inhibits macropinocytosis by lowering submembranous pH and preventing Rac1 and Cdc42 signaling. J. Cell Biol..

[40] Gomes dos Reis L., Lee W.H., Svolos M., Moir L.M., Jaber R., Engel A., Windhab N., Young P.M., Traini D. (2020). Delivery of pDNA to lung epithelial cells using PLGA nanoparticles formulated with a cell-penetrating peptide: Understanding the intracellular fate. Drug Dev. Ind. Pharm..

[41] Fittipaldi A., Ferrari A., Zoppé M., Arcangeli C., Pellegrini V., Beltram F., Giacca M. (2003). Cell Membrane Lipid Rafts Mediate Caveolar Endocytosis of HIV-1 Tat Fusion Proteins. J. Biol. Chem..

[42] Johnson J.R., Kocher B., Barnett E.M., Marasa J., Piwnica-Worms D. (2012). Caspase-activated cell-penetrating peptides reveal temporal coupling between endosomal release and apoptosis in an RGC-5 cell model. Bioconjug. Chem..

[43] Nolasco S., Bellido J., Serna M., Carmona B., Soares H., Zabala J.C. (2021). Colchicine Blocks Tubulin Heterodimer Recycling by Tubulin Cofactors TBCA, TBCB, and TBCE. Front. Cell Dev. Biol..

[44] He Z., Liu K., Manaloto E., Casey A., Cribaro G.P., Byrne H.J., Tian F., Barcia C., Conway G.E., Cullen P.J. (2018). Cold Atmospheric Plasma Induces ATP-Dependent Endocytosis of Nanoparticles and Synergistic U373MG Cancer Cell Death. Sci. Rep..

[45] Gross E., Bedlack R.S., Loew L.M. (1994). Dual-wavelength ratiometric fluorescence measurement of the membrane dipole potential. Biophys. J..

[46] Batta G., Kárpáti L., Henrique G.F., Tóth G., Tarapcsák S., Kovacs T., Zakany F., Mándity I.M., Nagy P. (2021). Statin-boosted cellular uptake and endosomal escape of penetratin due to reduced membrane dipole potential. Br. J. Pharmacol..

[47] Lättig-Tünnemann G., Prinz M., Hoffmann D., Behlke J., Palm-Apergi C., Morano I., Herce H.D., Cardoso M.C. (2011). Backbone rigidity and static presentation of guanidinium groups increases cellular uptake of arginine-rich cell-penetrating peptides. Nat. Commun..

[48] Li H., Zhang Y., Chen S.W., Li F.J., Zhuang S.M., Wang L.P., Zhang J., Song M. (2014). Prognostic significance of Flotillin1 expression in clinically N0 tongue squamous cell cancer. Int. J. Clin. Exp. Pathol..

[49] Pang L., Yang S., Dai W., Wu S., Kong J. (2022). Role of caveolin-1 in human organ function and disease: Friend or foe?. Carcinogenesis.

[50] Richard J.P., Melikov K., Brooks H., Prevot P., Lebleu B., Chernomordik L.V. (2005). Cellular uptake of unconjugated TAT peptide involves clathrin-dependent endocytosis and heparan sulfate receptors. J. Biol. Chem..

[51] Kaplan I.M., Wadia J.S., Dowdy S.F. (2005). Cationic TAT peptide transduction domain enters cells by macropinocytosis. J. Control. Release.

[52] Tanaka G., Nakase I., Fukuda Y., Masuda R., Oishi S., Shimura K., Kawaguchi Y., Takatani-Nakase T., Langel Ü., Gräslund A. (2012). CXCR4 stimulates macropinocytosis: Implications for cellular uptake of arginine-rich cell-penetrating peptides and HIV. Chem. Biol..

[53] Kawaguchi Y., Takeuchi T., Kuwata K., Chiba J., Hatanaka Y., Nakase I., Futaki S. (2016). Syndecan-4 Is a Receptor for Clathrin-Mediated Endocytosis of Arginine-Rich Cell-Penetrating Peptides. Bioconjug. Chem..

[54] Cleal K., He L., Watson P.D., Jones A.T. (2013). Endocytosis, Intracellular Traffic and Fate of Cell Penetrating Peptide Based Conjugates and Nanoparticles. Curr. Pharm. Des..

[55] Shin J.S., Abraham S.N. (2001). Caveolae as portals of entry for microbes. Microbes Infect..

[56] Gawande M., Branco P. (2011). An efficient and expeditious Fmoc protection of amines and amino acids in aqueous media. Green Chem..

[57] Bánóczi Z., Peregi B., Orbán E., Szabó R., Hudecz F. (2008). Synthesis of daunomycin-oligoarginine conjugates and their effect on human leukemia cells (HL-60). Arkivoc.

