# Supplementary material for: Cell-Penetrating Dabcyl-Containing Tetraarginines with Backbone Aromatics as Uptake Enhancers
Source: Pharmaceutics. 2022 Dec 31;15(1):141. doi: 10.3390/pharmaceutics15010141 (PMC9864790; doi:10.3390/pharmaceutics15010141)
Supplement: Supplementary file 1 [file pharmaceutics-15-00141-s001.zip › pharmaceutics-2118369-supplementary.pdf]

pharmaceutics

MDPI

Supplementary Materials: Cell-Penetrating Dabcyl-Containing Tetraarginines with Backbone Aromatics as Uptake Enhancers

Mo'ath Yousef, Ildikó Szabó, József Murányi, Françoise Illien, Dóra Soltész, Csaba Bató, Gabriella Tóth, Gyula Batta, Péter Nagy, Sandrine Sagan, Zoltán Bánóczi

1. Materials

All amino acid derivatives, Fmoc protected amino acids, 4-aminobenzoic acid (PABA), N,N'-diisopropylcarbodiimide (DIC), and Rink-amide MBHA resin were purchased from IRIS BiotechGmbH (Marktredwitz, Germany). N,N-diisopropylethylamine (DIEA), 1.8-diazabicyclo[5.4.0]undec-7-ene (DBU), thioanisole, 1,2-ethandithiol (EDT) were FLUKA (Buchs, Switzerland). Solvents for synthesis and purification were obtained from Molar Chemicals Ltd (Budapest, Hungary). 1-hydroxybenzotriazole (HOBt), phenol, 9-fluorenylmethoxycarbonyl chloride (Fmoc-Cl), 5(6)-carboxyfluorescein (Cf), 4-aminomethyl benzoic acid (AMBA), N-α-fmoc-N-ε-4-methyltrityl-lysine (Lys(Mtt)), trifluoroacetic acid (TFA), sodium azide (NaN_3), 2-deoxy-D-glucose (DOG), colchicine (COL) and all other chemicals used in biological experiments were purchased from Sigma Aldrich (Hungary). While tris(hydroxymethyl)aminomethane (TRIS) was purchased from VWR (Hungary). 1-[bis(dimethylamino)methylene]-1H-1,2,3-triazolo[4,5-b] pyridinium 3-oxide hexafluorophosphate (HATU), chlorpromazine (CPZ), methyl-beta-cyclodextrin (CyD), and 6-amino-2-naphthalenecarboxylic acid (NAPH) were purchased from TCI chemicals. Dabcyl was purchased from AAT Bioquest and Cyanine 5 dye (CY5) NHS ester was purchased from Lumiprobe.

1.1. Fmoc protection of AMBA

Fmoc protection of AMBA was performed according to[1], with minor modifications. Briefly 2 g of AMBA, and 1.2 eq of Fmoc-Cl were added to 20 ml of water: ethanol 3:1 and mixed overnight. The reaction mixture was then acidified to pH 4-5, extracted with ethyl acetate twice. The collected organic layers were then dried with sodium sulfate (Na_2SO_4), and the product was obtained after evaporation. The product was used without further purification (Yield 84%).

1.2. Synthesis of succinylated daunomycin (DauSuc)

Inclusion of the succinyl linker into daunomycin was performed in a method reported earlier.[2] 100 mg daunomycin×HCl was reacted with 2 eq succinic anhydride in the presence of 3 eq DIEA, the reactants were allowed to mix overnight in 5 ml DMF. The solvent was subsequently evaporated, and the remaining crude product was dissolved in mobile phase B (0.1%TFA in 80% ACN:20% H_2O) and further purified with RP-HPLC on a C18 Column.

1.3. RP-HPLC

[1] 1. M.B. Gawande, P.S. Branco, An efficient and expeditious Fmoc protection of amines and amino acids in aqueous media, Green Chem. 13 (2011) 3355–3359. https://doi.org/10.1039/C1GC15868F.

[2] Z. Bánóczi, B. Peregi, E. Orbán, R. Szabó, F. Hudecz, Synthesis of daunomycin-oligoarginine conjugates and their effect on human leukemia cells (HL-60), Arkivoc. 2008 (2008) 140–153. https://doi.org/10.3998/ark.5550190.0009.313.

Analytical RP-HPLC was performed on Exformma (Exformma Technology (ASIA) Co., Ltd, Hong Kong, China) HPLC system. The used columns were either Hypersil Hypurity C18 column (4.6 mm × 150 mm, 5 µm, 190 Å), or YMC ODS-A C18 column (4.6 mm × 150 mm, 3µm, 100 Å). Linear gradient elution (0 min 0% B; 2 min 0% B; 22 min 90% B) was used with eluent A (0.1% TFA in water) and eluent B (0.1% TFA in acetonitrile-water (80:20, *v*/*v*)) at 1mL/min flowrate, the peaks were detected at λ= 220 nm for both analytical and preparative RP-HPLC. The samples were dissolved in a minimum amount of eluent B and injected into the analytical RP-HPLC. The crude products were purified on a semi-preparative Phenomenex Jupiter C18 column (250 × 10 mm I.D.) with 10 mm silica (300 Å pore size) (Torrance, CA, USA). Flow rate was 4 mL/min. Linear gradient elution was applied. The samples were dissolved in eluent A containing small percent eluent B (10–25% depending on sequence).

1.4. Mass spectrometry

The molecular weight of peptides and conjugates was determined with ESI-MS. Using either Bruker Daltonics Esquire 3000 plus (Germany) ion trap mass spectrometer or Bruker Amazon SL (Germany). The samples were dissolved in water-acetonitrile solution (50:50) with 0.1% acetic acid. The samples were directly injected with a syringe pump. Parameters: capillary voltage: 4 kV, nebulizer gas: 10 psi, dry gas:4 L/min, heated capillary temperature: 250 °C.

1.5. Cell Culture

MCF-7 (ATCC: HTB-22) human breast adenocarcinoma and MDA-MB-231 (ATCC: HTB-26) human triple negative breast adenocarcinoma cells were used for the *in vitro* analysis. MCF-7 and MDA-MB-231 cells were maintained in Dulbecco's Modified Eagle Medium (DMEM) supplemented with 10% heat inactivated foetal calf serum (FCS), non-essential amino acids (NEAA), sodium pyruvate (1 mM), L-Glutamine (2 mM), 1% non-essential amino acids and 1% penicillin-streptomycin (from 10,000 units penicillin and 10mg/ml streptomycin). Cells were maintained in plastic tissue culture dishes at 37 °C with a humidified atmosphere containing 5% CO_2/95% air.

Head and neck squamous cell carcinoma cell lines Detroit 562 (CCL-138™), FaDu (HTB-43™) and SCC-25 (CRL-1628™) were obtained from American Type Culture Collection (ATCC). Detroit 562 cells were cultured in EMEM (Lonza) supplemented with 10% (*v*/*v*) fetal bovine serum (FBS, GIBCO), 0.1% (*v*/*v*) sodium pyruvate (Lonza) and 1% (*v*/*v*) antibiotic mix (MycoZap Plus-CL, Lonza). FaDu cells were maintained DMEM (Lonza) supplemented with 10% (*v*/*v*) fetal bovine serum (FBS, GIBCO), 0.1% added sodium pyruvate (Lonza) and 1% antibiotic mix (MycoZap Plus-CL, Lonza). SCC25 cells were cultured DMEM:F12 (Lonza) supplemented with 10% (*v*/*v*) fetal bovine serum (FBS, GIBCO), 400 ng/mL hydrocortisone (STEMCELL) and 1% antibiotic mix (MycoZap Plus-CL, Lonza) respectively in humidified atmosphere at 37 °C and 5% CO_2. Cells were checked for mycoplasma (MycoAlert™ PLUS Mycoplasma Detection Kit, Cat. No. LT07-705, Lonza, Basel, Switzerland). Paclitaxel (Cat. No.S1150) was purchased from Selleckchem (Houston, TX, USA).

2. Fluorimetry

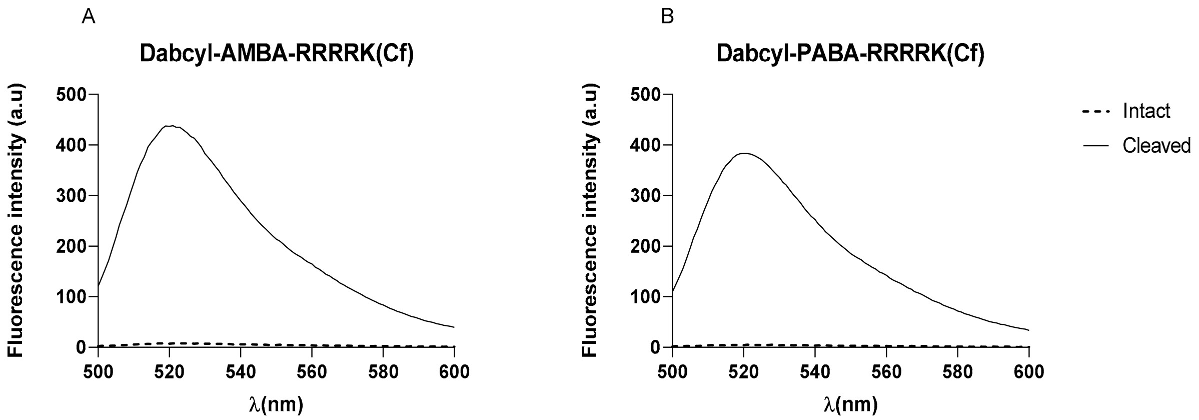

Figure S1. The effect of cleavage of peptides by trypsin on fluorescence intensity. Conjugates **A)** Dabcyl-AMBA-RRRRK(Cf) and **B)** Dabcyl-AMBA-RRRRK(Cf) were digested by trypsin at 2.5 μM concentration. The fluorescence of peptides was recorded in the presence and absence of trypsin after 15 min. Ex. Wavelength (nm) 480nm, emission 500-600 nm, voltage 500 V, instrument: Cary Eclipse.

3. Time dependence of internalized peptides

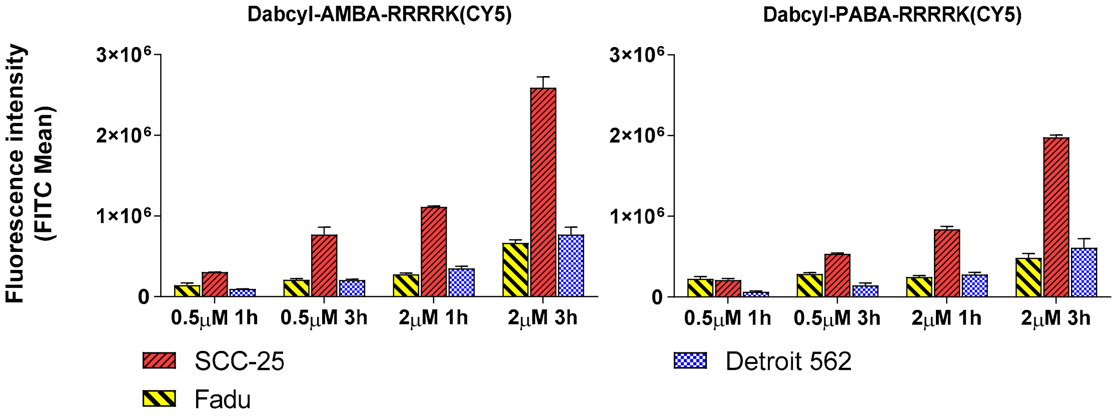

Figure S2. Time dependence of peptides on FADU, SCC-25, and Detroit 562 cells. Cells were treated with 0.5 and 2μM peptide solutions for 60 mins. The fluorescence intensity of the cells was determined by flow cytometry after trypsin treatment.

4. The effect of membrane potential

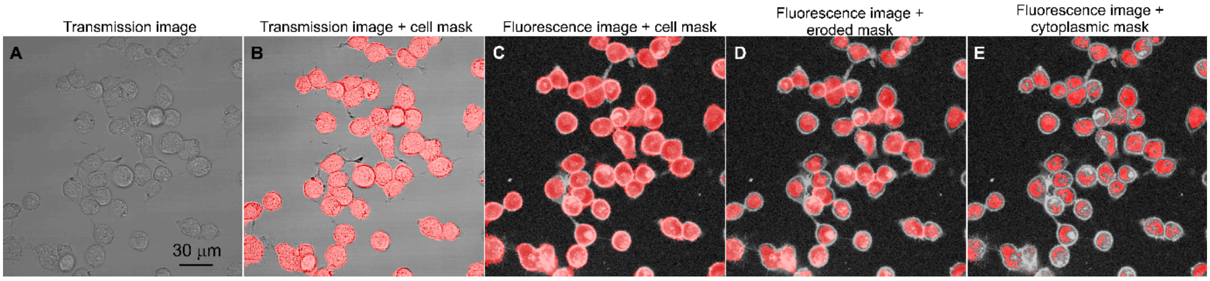

Figure S3. Image segmentation for identification of cells and the cytoplasm. Cells were outlined based on transmission images (A). The cellular mask was created by a convolutional neural network, and it is shown overlaid on the transmission image (B) and the fluorescence image (C). The mask was eroded to remove edge pixels corresponding to the membrane (D). A variance filter was

applied to this eroded mask, and pixels surrounded by a neighborhood with a high variance, corresponding to endosomes, were removed from the mask (E). The threshold variance above which pixels were deleted from the mask was determined by visual inspection. This final mask corresponds to the cytoplasm.

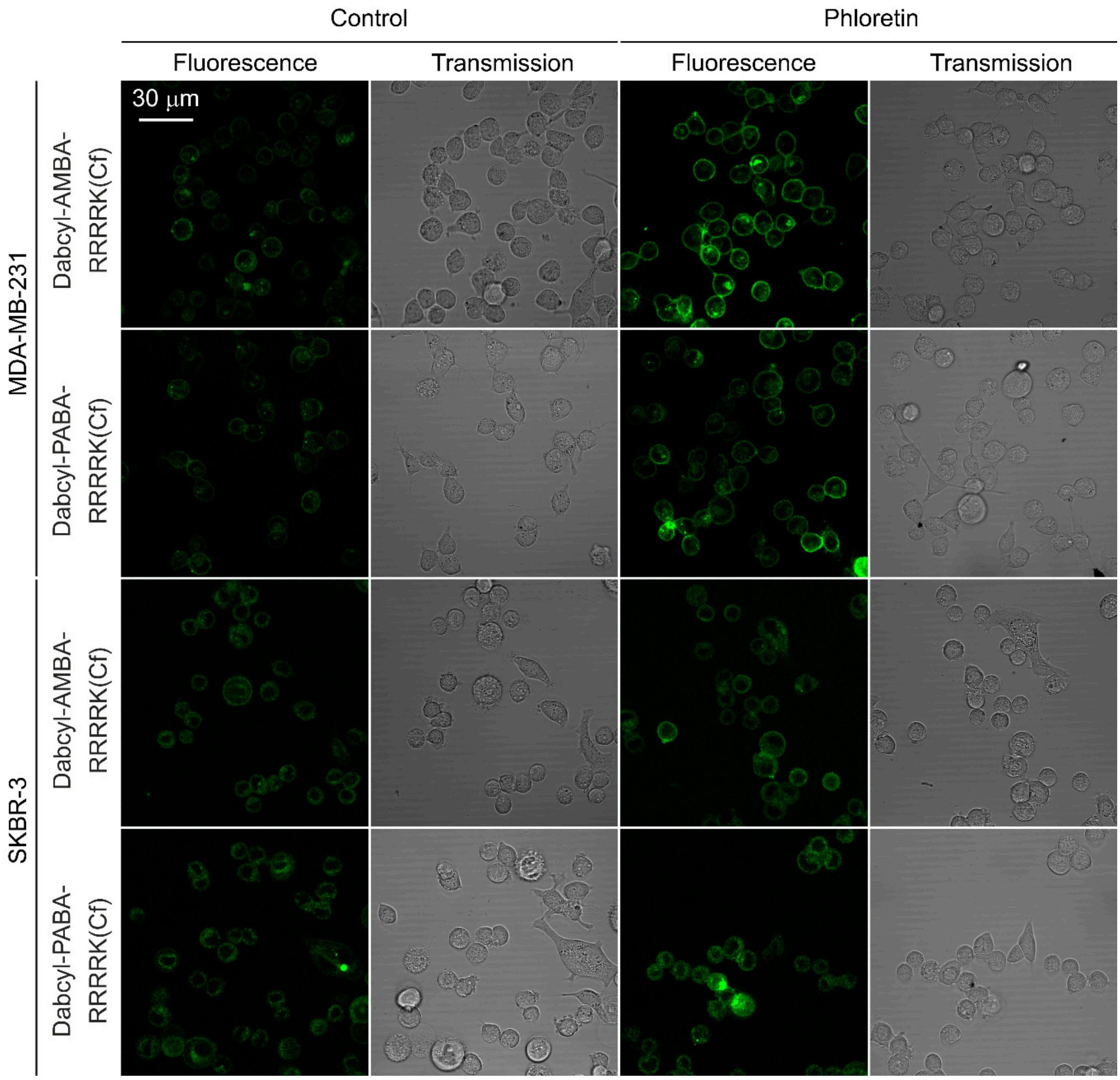

Figure S4. Representative microscopic images showing the uptake of peptides and the effect of the diminished dipole potential. MDA-MB-231 and SKBR-3 cells were pretreated with 100 µM phloretin in the presence of 0.05% (*v*/*v*) Pluronic F-127 at room temperature for 60 min in order to decrease their dipole potential. Control samples were only incubated with Pluronic F-127. Preincubation was followed by treating the cells with 5 µM of Dabcyl-AMBA-RRRRK(Cf) or Dabcyl-PABA-RRRRK(Cf) for 20 minutes followed by taking confocal microscopic images. Images showing the fluorescence of the peptides and the corresponding transmission images are shown in the figure. The scale bar applies to all images.

5. Chemical Characterization of Peptide Conjugates

5.1. RP-HPLC

Analytical RP-HPLC was measured on Exformma (Exformma Technology (ASIA) Co., Ltd, Hong Kong, China) HPLC system. The peptide conjugates were injected on Hypersil Hypurity C18 column (4.6 mm × 150 mm, 5 µm, 190 Å). In some cases, YMC ODS-A C18 column (4.6 mm × 150 mm, 3µm, 100 Å) was also used. Linear gradient elution (0 min 0% B; 2 min 0% B; 22 min 90% B) was used with eluent A (0.1% TFA in water) and

eluent B (0.1% TFA in acetonitrile-water (80:20, *v*/*v*)) at 1mL/min flowrate, the peaks were detected at λ= 220 nm for both analytical and preparative RP-HPLC. The samples were dissolved in a minimum amount of eluent B and injected into the analytical RP-HPLC.

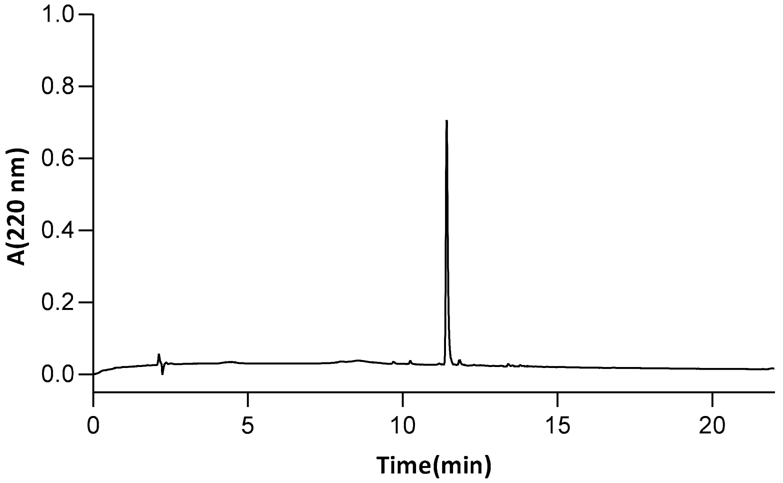

Figure S5. HPLC chromatogram of Cf-Arg8. Retention time was obtained on YMC ODS-A C18 column (4.6 mm × 150 mm, 3μm, 100 Å). The applied linear gradient elution was 0 min 0% B, 2 min 0% B, 22 min 90% B at 1 mL/min flow rate. The detection was carried on at λ = 220 nm.

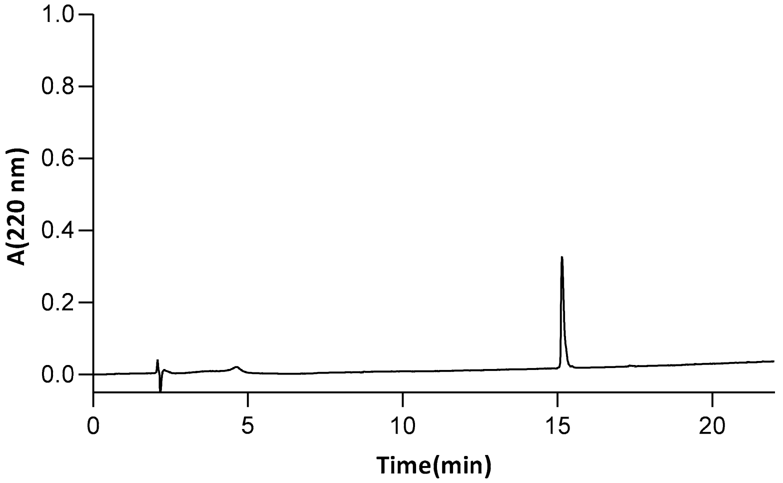

Figure S6. HPLC chromatogram of Cy5-Arg8. Retention time was obtained on Hypersil Hypurity C18 column (4.6 mm × 150 mm, 5 μm, 190 Å). The applied linear gradient elution was 0 min 0% B, 2 min 0% B, 22 min 90% B at 1 mL/min flow rate. The detection was carried on at λ = 220 nm.

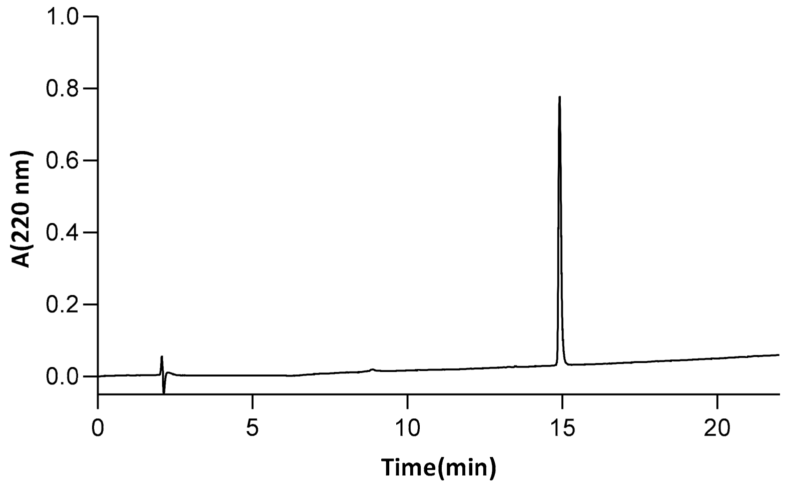

Figure S7. HPLC chromatogram of Dabcyl-AMBA-RRRRK(Cf). Retention time was obtained on Hypersil Hypurity C18 column (4.6 mm × 150 mm, 5 µm, 190 Å). The applied linear gradient elution was 0 min 0% B, 2 min 0% B, 22 min 90% B at 1 mL/min flow rate. The detection was carried on at λ = 220 nm.

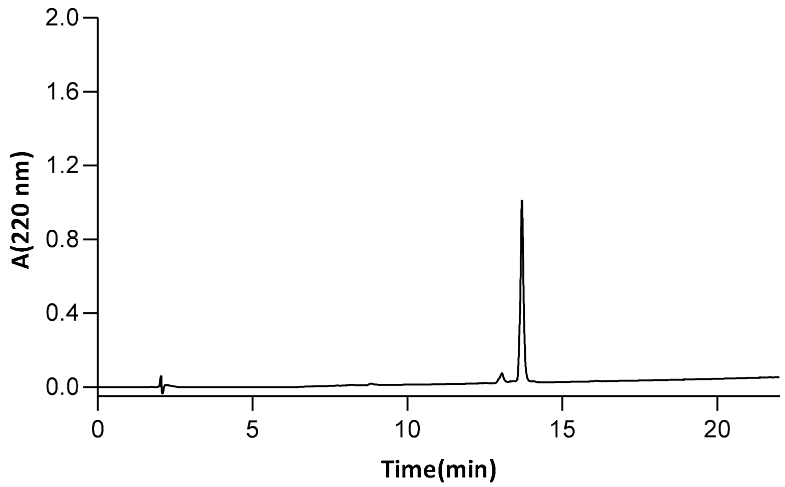

Figure S8. HPLC chromatogram of Dabcyl-RR-AMBA-RRK(Cf). Retention time was obtained on Hypersil Hypurity C18 column (4.6 mm × 150 mm, 5 µm, 190 Å). The applied linear gradient elution was 0 min 0% B, 2 min 0% B, 22 min 90% B at 1 mL/min flow rate. The detection was carried on at λ = 220 nm.

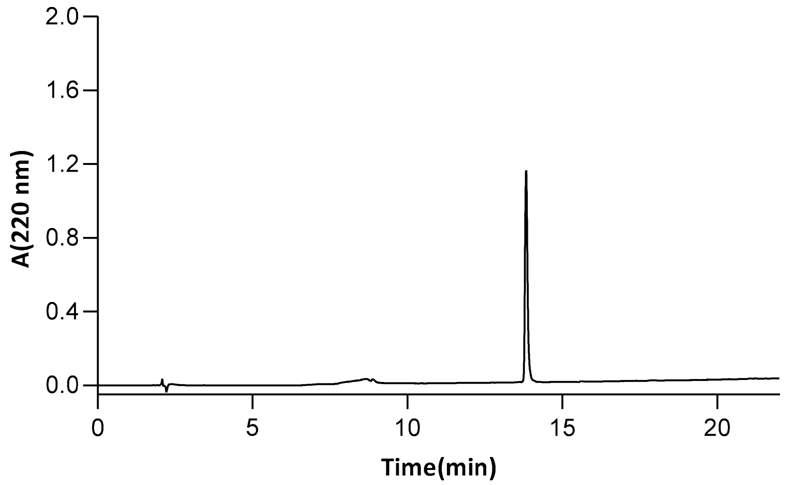

Figure S9. HPLC chromatogram of Dabcyl-RR-AMBA-RR-AMBA-K(Cf). Retention time was obtained on Hypersil Hypurity C18 column (4.6 mm × 150 mm, 5 µm, 190 Å). The applied linear gradient elution was 0 min 0% B, 2 min 0% B, 22 min 90% B at 1 mL/min flow rate. The detection was carried on at λ = 220 nm.

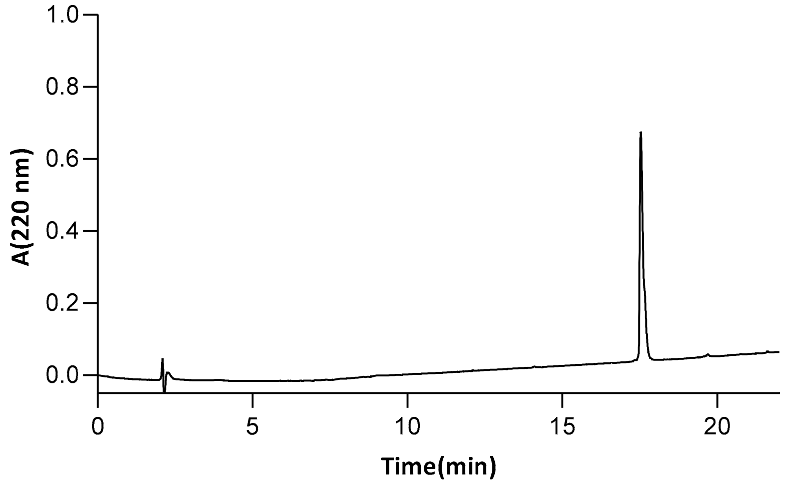

Figure S10. HPLC chromatogram of Dabcyl-AMBA-RRRRK(Cy5). Retention time was obtained on Hypersil Hypurity C18 column (4.6 mm × 150 mm, 5 µm, 190 Å). The applied linear gradient elution was 0 min 0% B, 2 min 0% B, 22 min 90% B at 1 mL/min flow rate. The detection was carried on at λ = 220 nm.

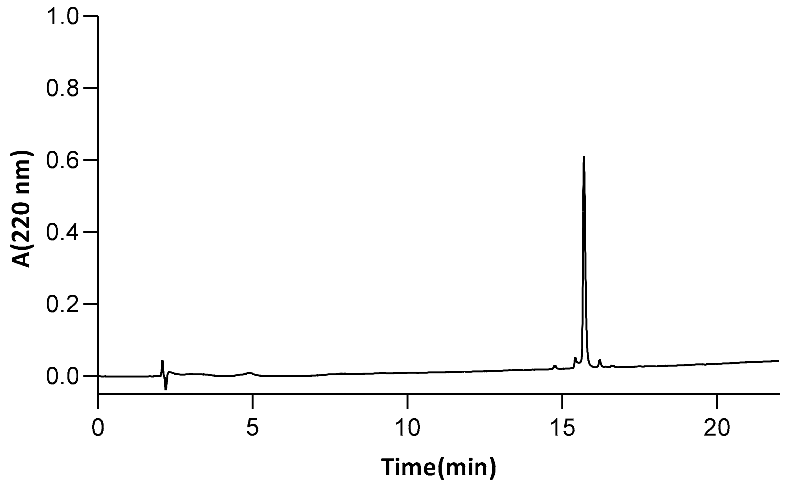

Figure S11. HPLC chromatogram of Dabcyl-AMBA-RRRRK(DauSuc). Retention time was obtained on Hypersil Hypurity C18 column (4.6 mm × 150 mm, 5 μm, 190 Å). The applied linear gradient elution was 0 min 0% B, 2 min 0% B, 22 min 90% B at 1 mL/min flow rate. The detection was carried on at λ = 220 nm.

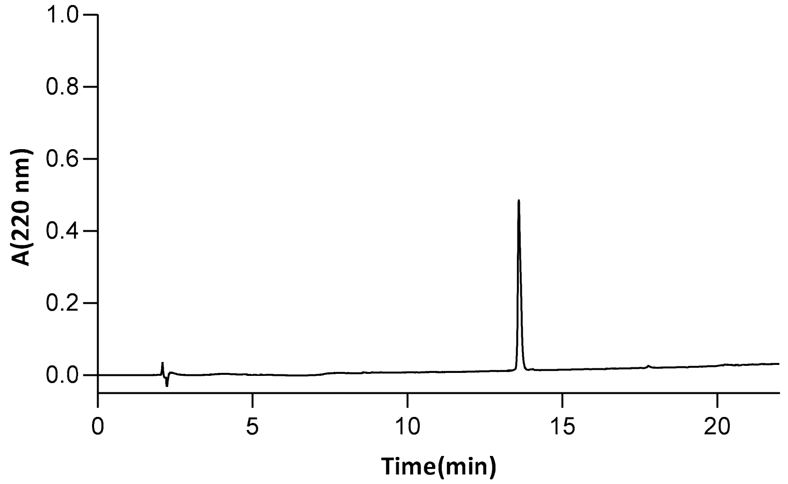

Figure S12. HPLC chromatogram of Dabcyl-AMBA-RRRRK(MTX). Retention time was obtained on Hypersil Hypurity C18 column (4.6 mm × 150 mm, 5 μm, 190 Å). The applied linear gradient elution was 0 min 0% B, 2 min 0% B, 22 min 90% B at 1 mL/min flow rate. The detection was carried on at λ = 220 nm.

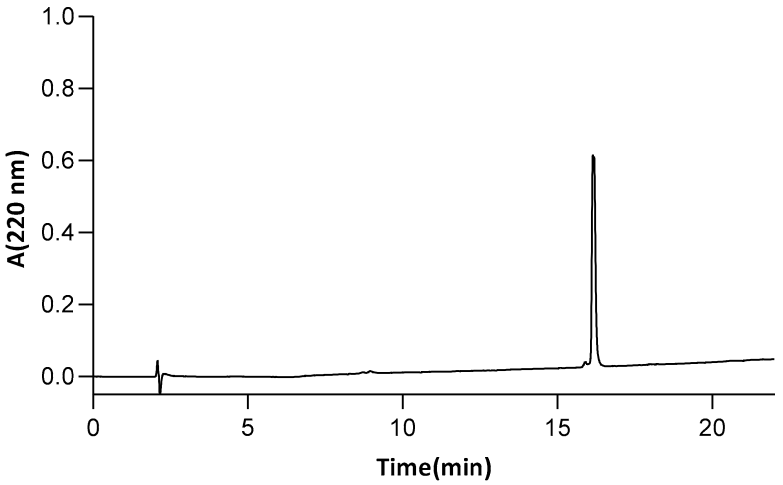

Figure S13. HPLC chromatogram of Dabcyl-NAPH-RRRRK(Cf). Retention time was obtained on Hypersil Hypurity C18 column (4.6 mm × 150 mm, 5 μm, 190 Å). The applied linear gradient elution was 0 min 0% B, 2 min 0% B, 22 min 90% B at 1 mL/min flow rate. The detection was carried on at λ = 220 nm.

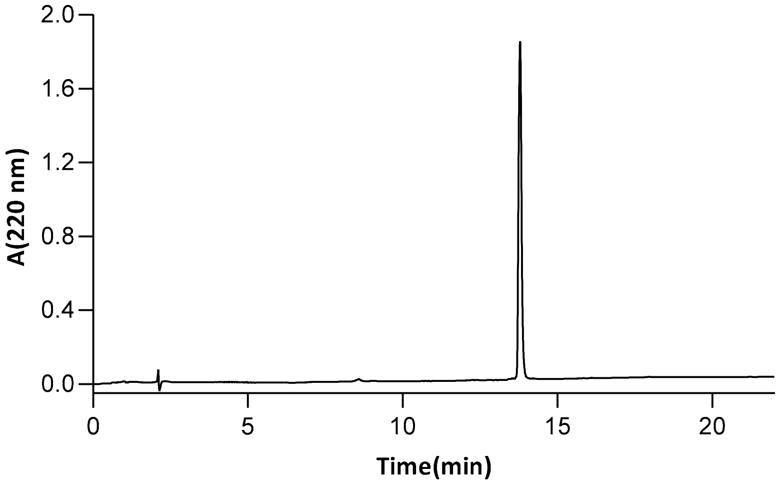

Figure S14. HPLC chromatogram of Dabcyl-RR-NAPH-RR-K(Cf). Retention time was obtained on Hypersil Hypurity C18 column (4.6 mm × 150 mm, 5 μm, 190 Å). The applied linear gradient elution was 0 min 0% B, 2 min 0% B, 22 min 90% B at 1 mL/min flow rate. The detection was carried on at λ = 220 nm.

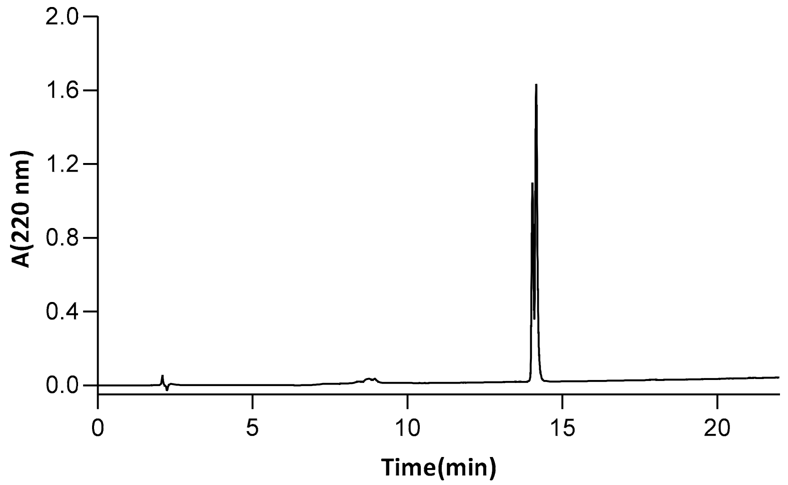

Figure S15. HPLC chromatogram of Dabcyl-RR-NAPH-RR-NAPH-K(Cf). Retention time was obtained on Hypersil Hypurity C18 column (4.6 mm × 150 mm, 5 μm, 190 Å). The applied linear gradient elution was 0 min 0% B, 2 min 0% B, 22 min 90% B at 1 mL/min flow rate. The detection was carried on at λ = 220 nm.

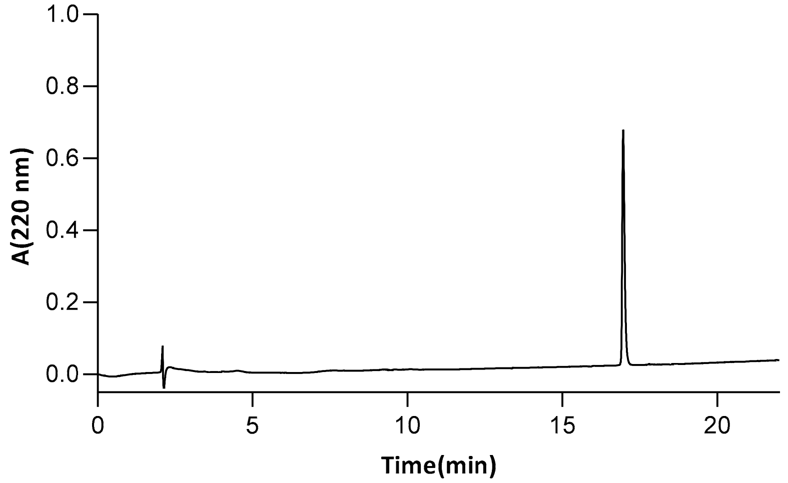

Figure S16. HPLC chromatogram of Dabcyl-NAPH-RRRR-K(DauSuc). Retention time was obtained on Hypersil Hypurity C18 column (4.6 mm × 150 mm, 5 μm, 190 Å). The applied linear gradient elution was 0 min 0% B, 2 min 0% B, 22 min 90% B at 1 mL/min flow rate. The detection was carried on at λ = 220 nm.

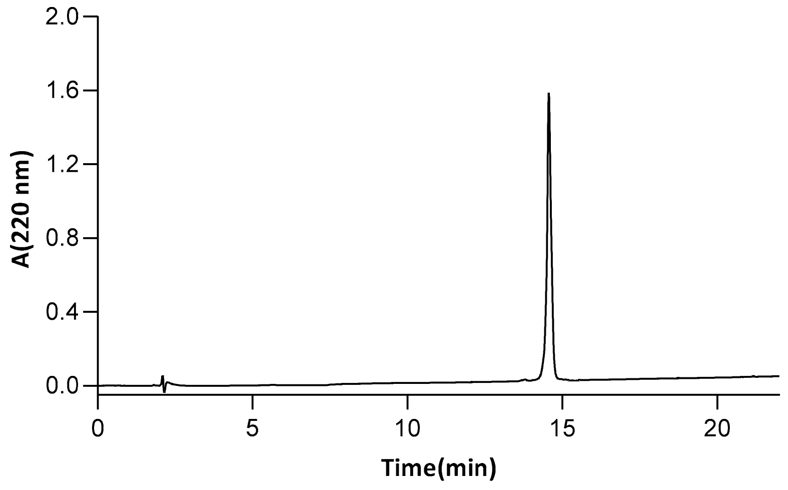

Figure S17. HPLC chromatogram of Dabcyl-NAPH-RRRR-K(MTX). Retention time was obtained on Hypersil Hypurity C18 column (4.6 mm × 150 mm, 5 μm, 190 Å). The applied linear gradient elution was 0 min 0% B, 2 min 0% B, 22 min 90% B at 1 mL/min flow rate. The detection was carried on at λ = 220 nm.

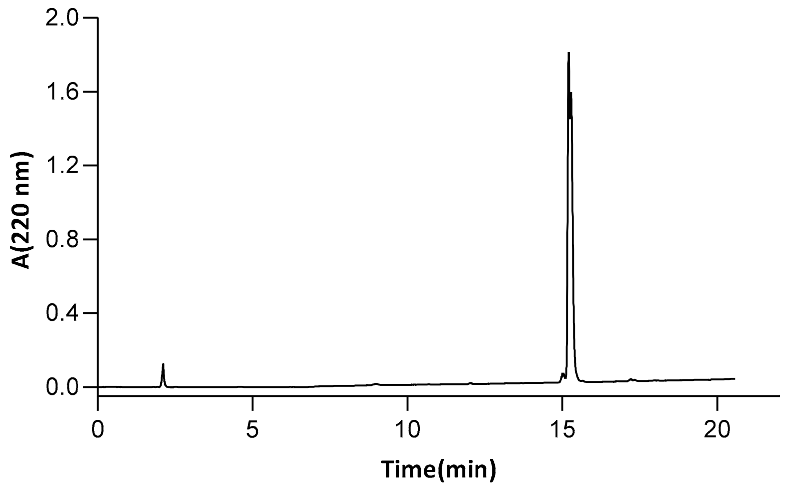

Figure S18. HPLC chromatogram of Dabcyl-PABA-RRRR-K(Cf). Retention time was obtained on Hypersil Hypurity C18 column (4.6 mm × 150 mm, 5 μm, 190 Å). The applied linear gradient elution was 0 min 0% B, 2 min 0% B, 22 min 90% B at 1 mL/min flow rate. The detection was carried on at λ = 220 nm.

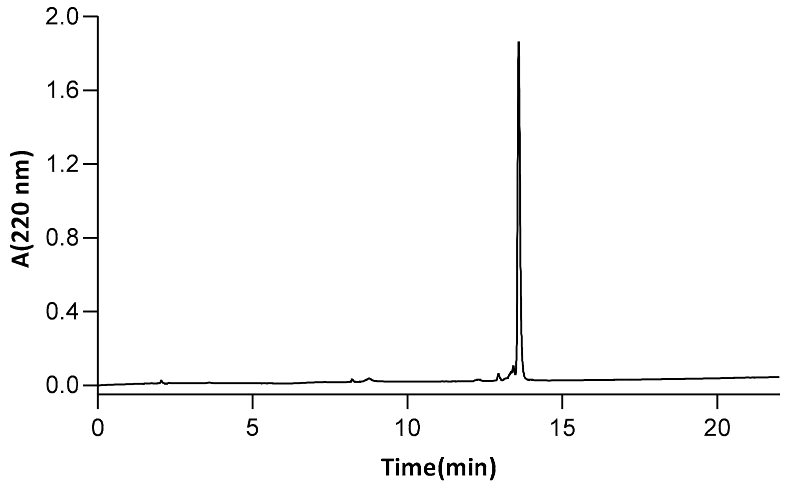

Figure S19. HPLC chromatogram of Dabcyl-RR-PABA-RR-K(Cf). Retention time was obtained on Hypersil Hypurity C18 column (4.6 mm × 150 mm, 5 μm, 190 Å). The applied linear gradient elution was 0 min 0% B, 2 min 0% B, 22 min 90% B at 1 mL/min flow rate. The detection was carried on at λ = 220 nm.

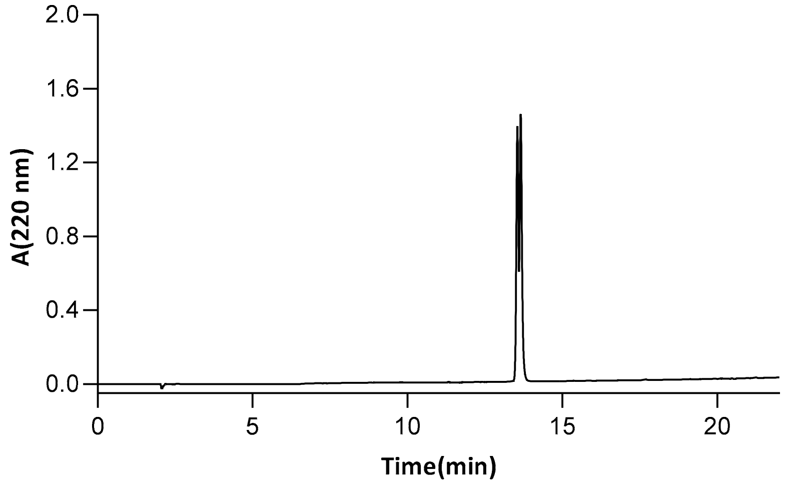

Figure S20. HPLC chromatogram of Dabcyl-RR-PABA-RR-PABA-K(Cf). Retention time was obtained on Hypersil Hypurity C18 column (4.6 mm × 150 mm, 5 μm, 190 Å). The applied linear gradient elution was 0 min 0% B, 2 min 0% B, 22 min 90% B at 1 mL/min flow rate. The detection was carried on at λ = 220 nm.

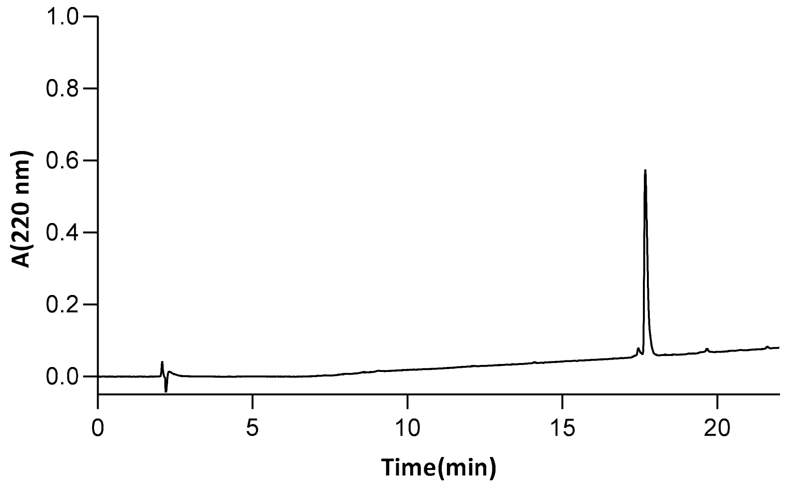

Figure S21. HPLC chromatogram of Dabcyl-PABA-RRRR-K(Cy5). Retention time was obtained on Hypersil Hypurity C18 column (4.6 mm × 150 mm, 5 μm, 190 Å). The applied linear gradient elution was 0 min 0% B, 2 min 0% B, 22 min 90% B at 1 mL/min flow rate. The detection was carried on at λ = 220 nm.

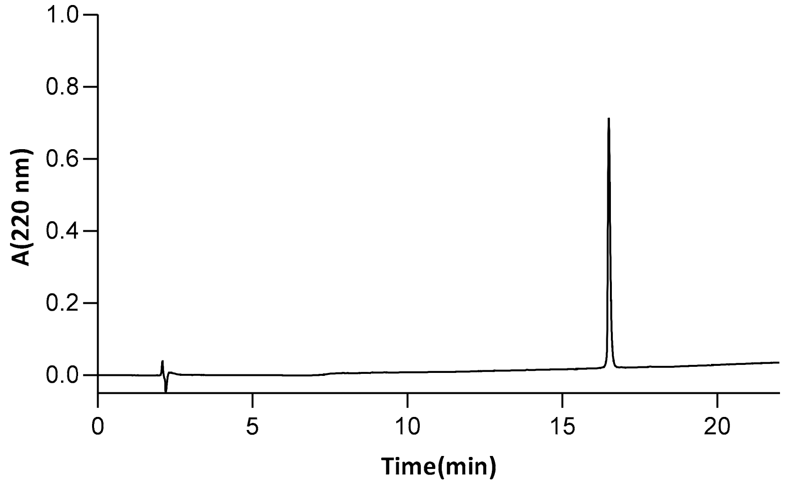

Figure S22. HPLC chromatogram of Dabcyl-PABA-RRRR-K(DauSuc). Retention time was obtained on Hypersil Hypurity C18 column (4.6 mm × 150 mm, 5 μm, 190 Å). The applied linear gradient elution was 0 min 0% B, 2 min 0% B, 22 min 90% B at 1 mL/min flow rate. The detection was carried on at λ = 220 nm.

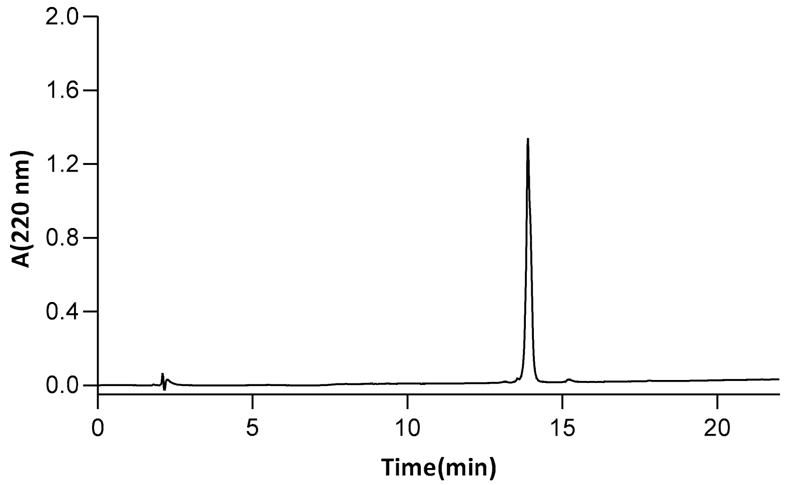

Figure S23. HPLC chromatogram of Dabcyl-PABA-RRRR-K(MTX). Retention time was obtained on Hypersil Hypurity C18 column (4.6 mm × 150 mm, 5 μm, 190 Å). The applied linear gradient elution was 0 min 0% B, 2 min 0% B, 22 min 90% B at 1 mL/min flow rate. The detection was carried on at λ = 220 nm.

5.2. Mass spectrometry

The molecular weight of peptide conjugates was determined with ESI-MS. Using either Bruker Daltonics Esquire 3000 plus (Germany) ion trap mass spectrometer or Bruker Amazon SL (Germany). The samples were dissolved in water-acetonitrile solution (50:50) with 0.1% acetic acid. The samples were directly injected with a syringe pump. Parameters: capillary voltage: 4 kV, nebulizer gas: 10 psi, dry gas:4 L/min, heated capillary temperature: 250 ºC.

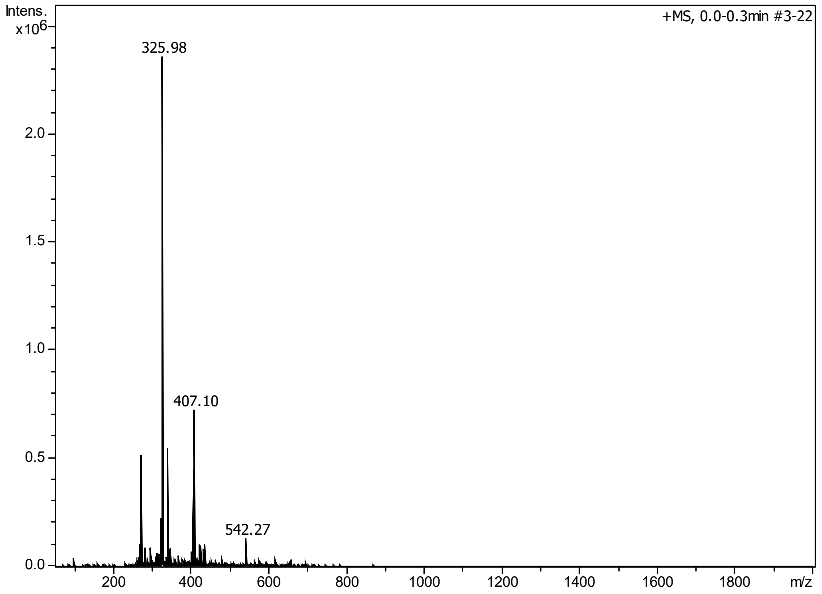

Figure S24. MS Spectrum of Cf-Arg8. The identity of the peptide conjugate was determined using Bruker Daltonics Esquire 3000 plus (Germany) ion trap mass spectrometer. The samples are dissolved in water-acetonitrile (50:50) with 0.1% acetic acid.

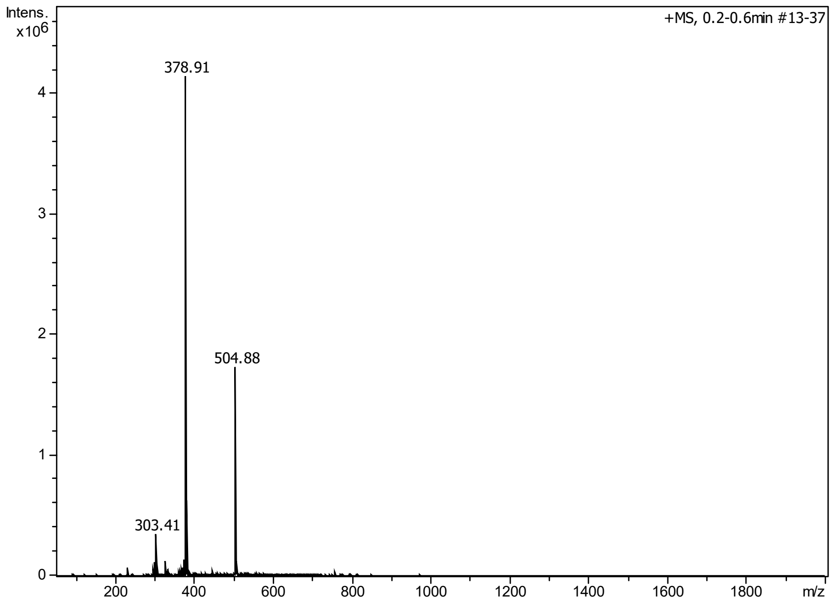

Figure S25. MS Spectrum of Dabcyl-AMBA-RRRRK(Cf). The identity of the peptide conjugate was determined using Bruker Daltonics Esquire 3000 plus (Germany) ion trap mass spectrometer. The samples are dissolved in water-acetonitrile (50:50) with 0.1% acetic acid.

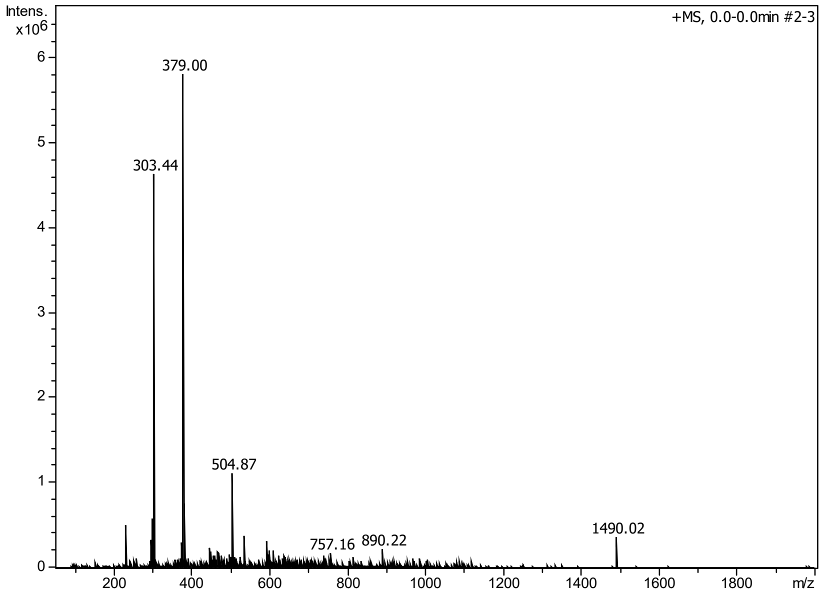

Figure S26. MS Spectrum of Dabcyl-RR-AMBA-RRK(Cf). The identity of the peptide conjugate was determined using Bruker Daltonics Esquire 3000 plus (Germany) ion trap mass spectrometer. The samples are dissolved in water-acetonitrile (50:50) with 0.1% acetic acid.

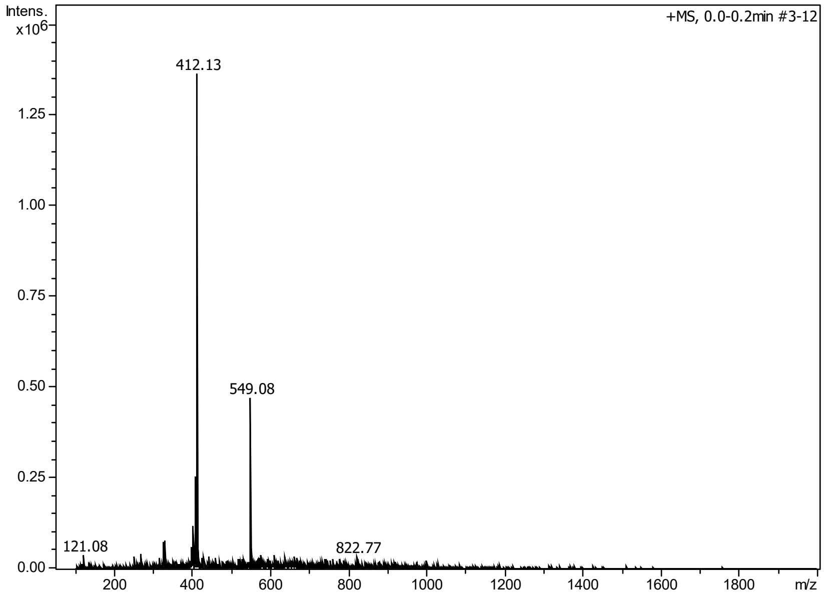

Figure S27. MS Spectrum of Dabcyl-RR-AMBA-RR-AMBA-K(Cf). The identity of the peptide conjugate was determined using Bruker Daltonics Esquire 3000 plus (Germany) ion trap mass spectrometer. The samples are dissolved in water-acetonitrile (50:50) with 0.1% acetic acid.

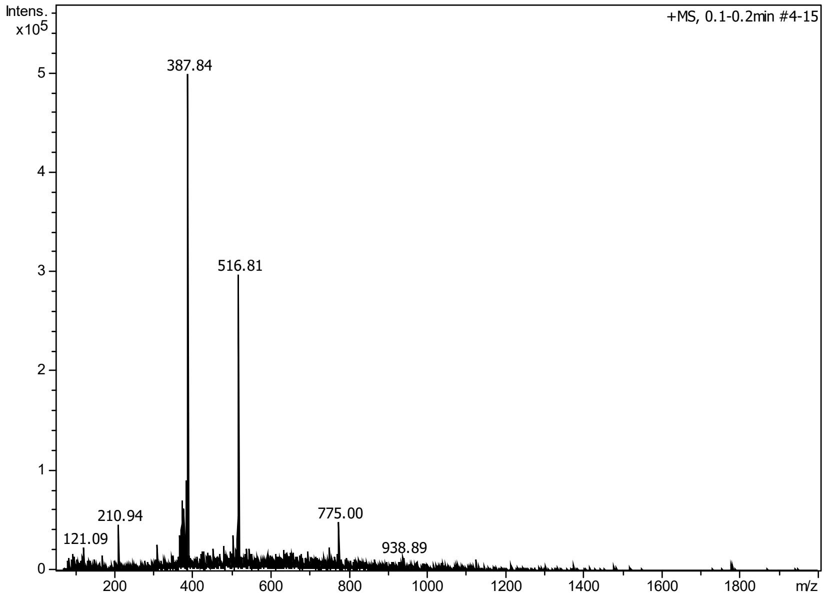

Figure S28. MS Spectrum of Dabcyl-NAPH-RRRRK(Cf). The identity of the peptide conjugate was determined using Bruker Daltonics Esquire 3000 plus (Germany) ion trap mass spectrometer. The samples are dissolved in water-acetonitrile (50:50) with 0.1% acetic acid.

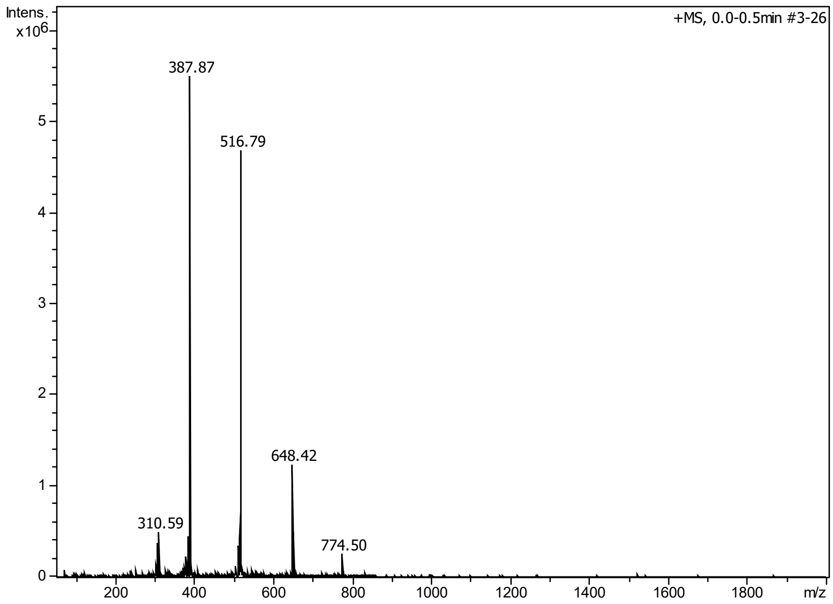

Figure S29. MS Spectrum of Dabcyl-RR-NAPH-RRK(Cf). The identity of the peptide conjugate was determined using Bruker Daltonics Esquire 3000 plus (Germany) ion trap mass spectrometer. The samples are dissolved in water-acetonitrile (50:50) with 0.1% acetic acid.

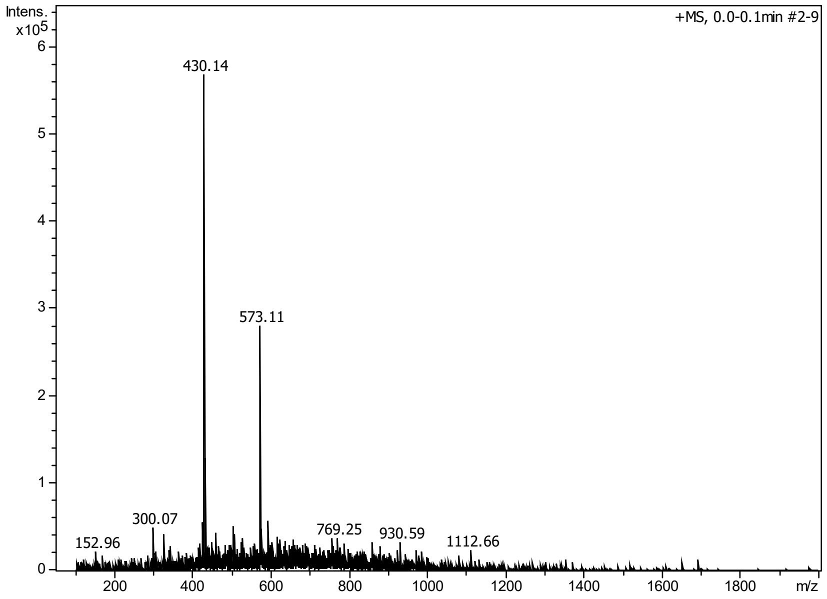

Figure S30. MS Spectrum of Dabcyl-RR-NAPH-RR-NAPH-K(Cf). The identity of the peptide conjugate was determined using Bruker Daltonics Esquire 3000 plus (Germany) ion trap mass spectrometer. The samples are dissolved in water-acetonitrile (50:50) with 0.1% acetic acid.

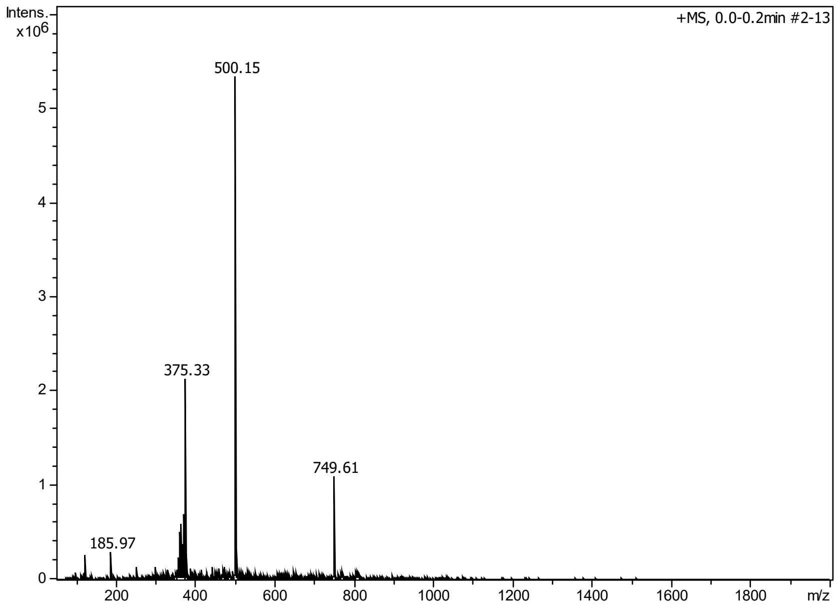

Figure S31. MS Spectrum of Dabcyl-PABA-RRRRK(Cf). The identity of the peptide conjugate was determined using Bruker Daltonics Esquire 3000 plus (Germany) ion trap mass spectrometer. The samples are dissolved in water-acetonitrile (50:50) with 0.1% acetic acid.

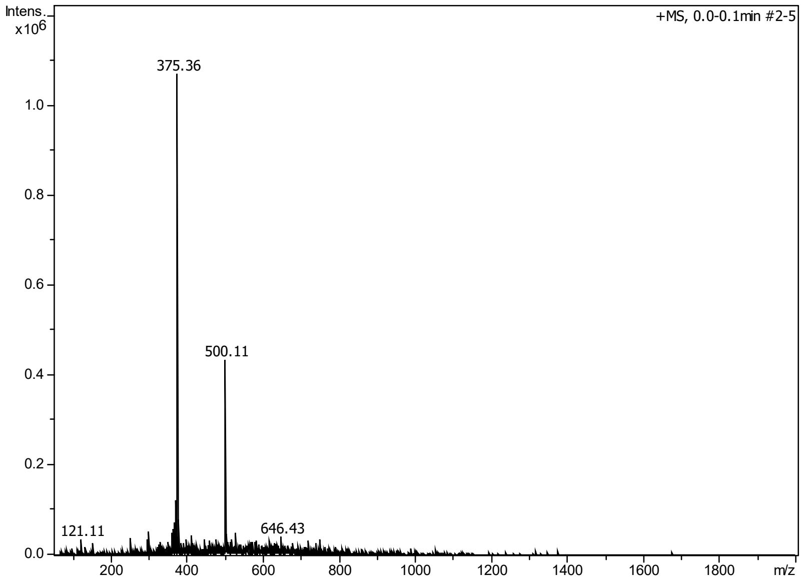

Figure S32. MS Spectrum of Dabcyl-RR-PABA-RRK(Cf). The identity of the peptide conjugate was determined using Bruker Daltonics Esquire 3000 plus (Germany) ion trap mass spectrometer. The samples are dissolved in water-acetonitrile (50:50) with 0.1% acetic acid.

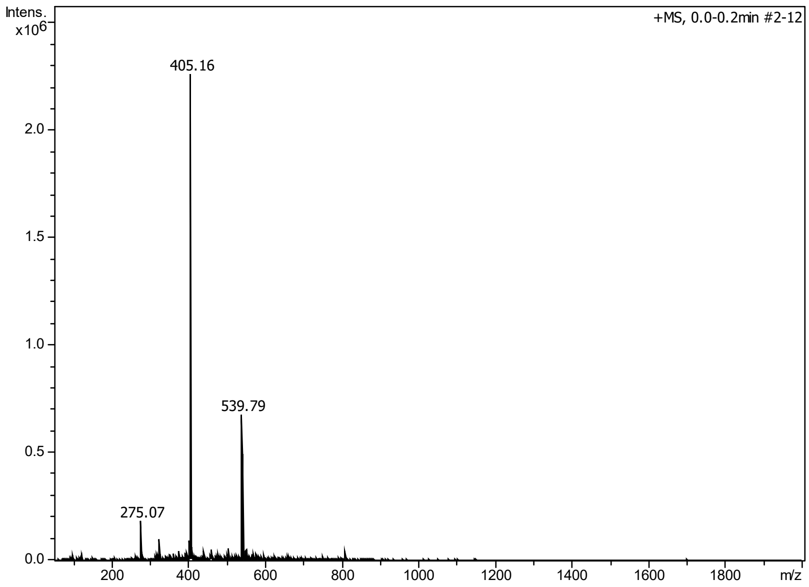

Figure S33. MS Spectrum of Dabcyl-RR-PABA-RR-PABA-K(Cf). The identity of the peptide conjugate was determined using Bruker Daltonics Esquire 3000 plus (Germany) ion trap mass spectrometer. The samples are dissolved in water-acetonitrile (50:50) with 0.1% acetic acid.

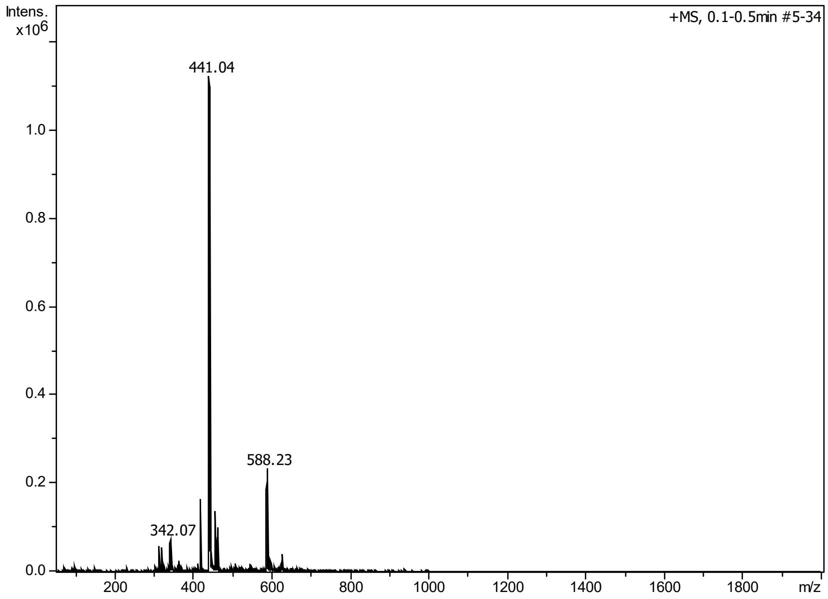

Figure S34. MS Spectrum of Dabcyl-AMBA-RRRRK(DauSuc). The identity of the peptide conjugate was determined using Bruker Daltonics Esquire 3000 plus (Germany) ion trap mass spectrometer. The samples are dissolved in water-acetonitrile (50:50) with 0.1% acetic acid.

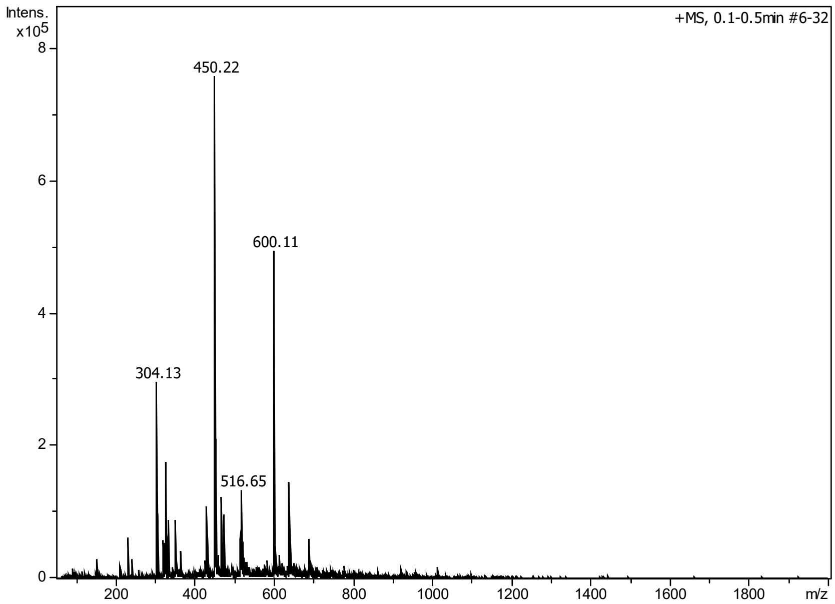

Figure S35. MS Spectrum of Dabcyl-NAPH-RRRRK(DauSuc). The identity of the peptide conjugate was determined using Bruker Daltonics Esquire 3000 plus (Germany) ion trap mass spectrometer. The samples are dissolved in water-acetonitrile (50:50) with 0.1% acetic acid.

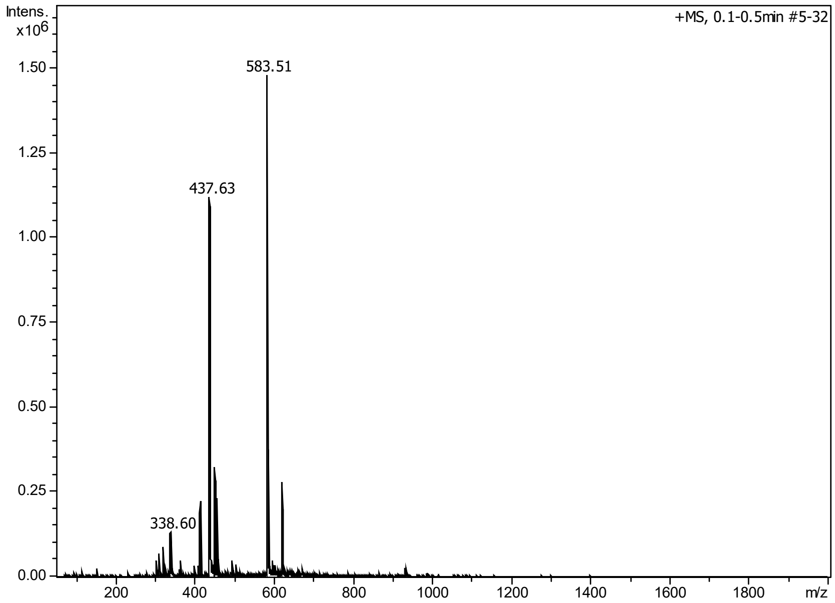

Figure S36. MS Spectrum of Dabcyl-PABA-RRRRK(DauSuc). The identity of the peptide conjugate was determined using Bruker Daltonics Esquire 3000 plus (Germany) ion trap mass spectrometer. The samples are dissolved in water-acetonitrile (50:50) with 0.1% acetic acid.

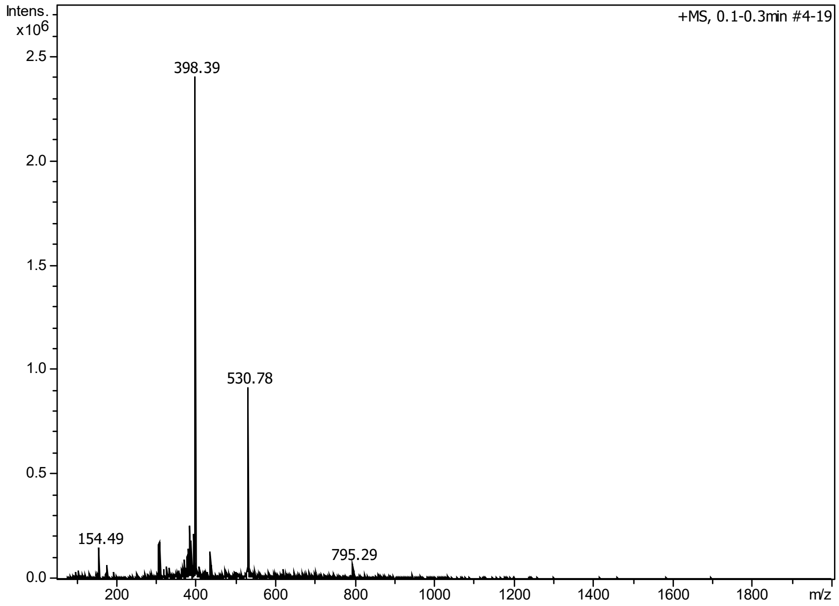

Figure S37. MS Spectrum of Dabcyl-AMBA-RRRRK(MTX). The identity of the peptide conjugate was determined using Bruker Daltonics Esquire 3000 plus (Germany) ion trap mass spectrometer. The samples are dissolved in water-acetonitrile (50:50) with 0.1% acetic acid.

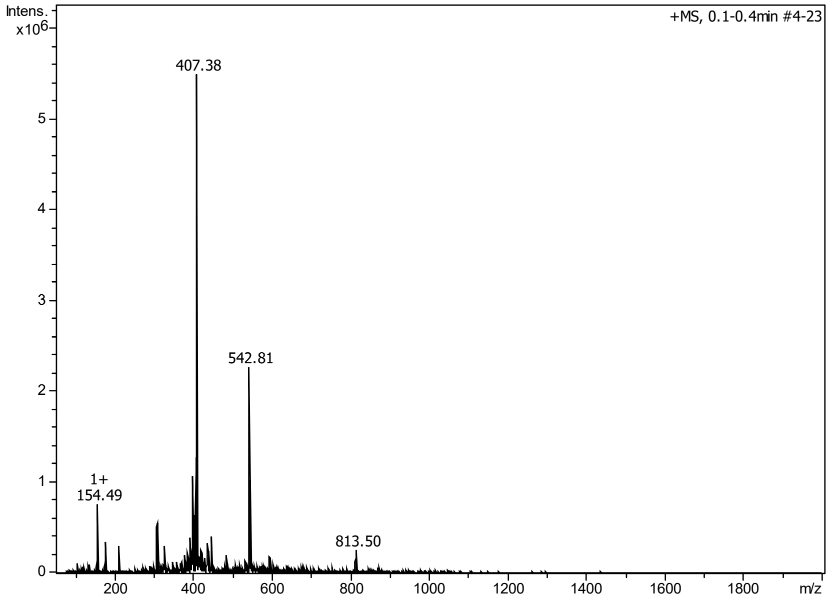

Figure S38. MS Spectrum of Dabcyl-NAPH-RRRRK(MTX). The identity of the peptide conjugate was determined using Bruker Daltonics Esquire 3000 plus (Germany) ion trap mass spectrometer. The samples are dissolved in water-acetonitrile (50:50) with 0.1% acetic acid.

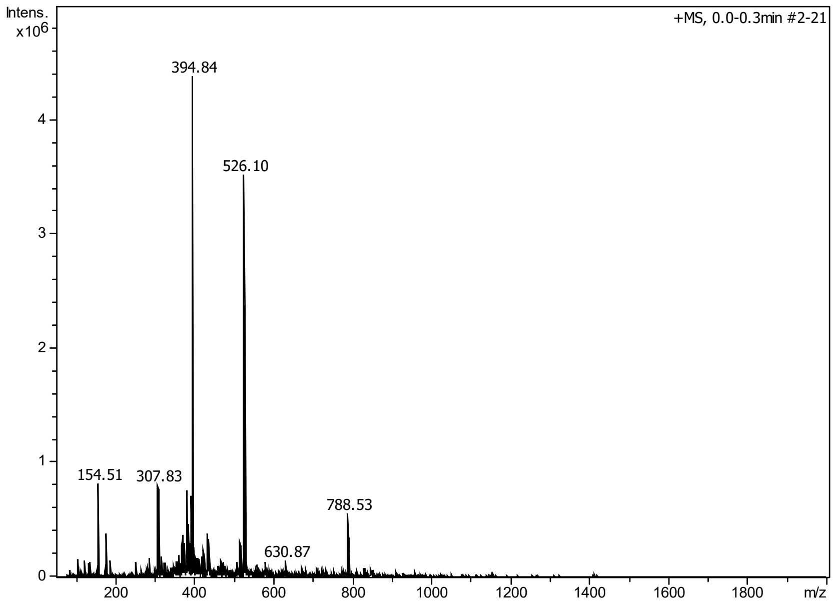

Figure S39. MS Spectrum of Dabcyl-PABA-RRRRK(MTX). The identity of the peptide conjugate was determined using Bruker Daltonics Esquire 3000 plus (Germany) ion trap mass spectrometer. The samples are dissolved in water-acetonitrile (50:50) with 0.1% acetic acid.

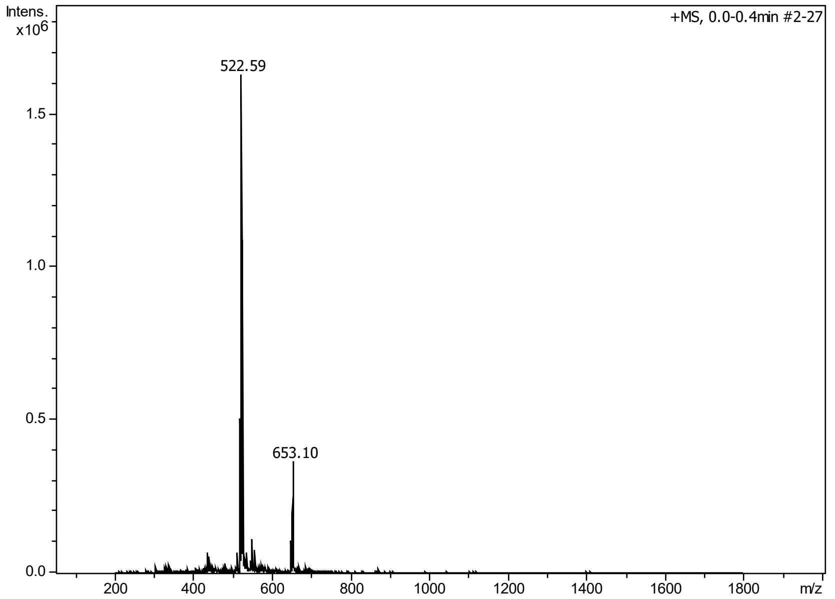

Figure S40. MS Spectrum of Dabcyl-AMBA-RRRRK(GFLG-E5-MTX). The identity of the peptide conjugate was determined using Bruker Daltonics Esquire 3000 plus (Germany) ion trap mass spectrometer. The samples are dissolved in water-acetonitrile (50:50) with 0.1% acetic acid.

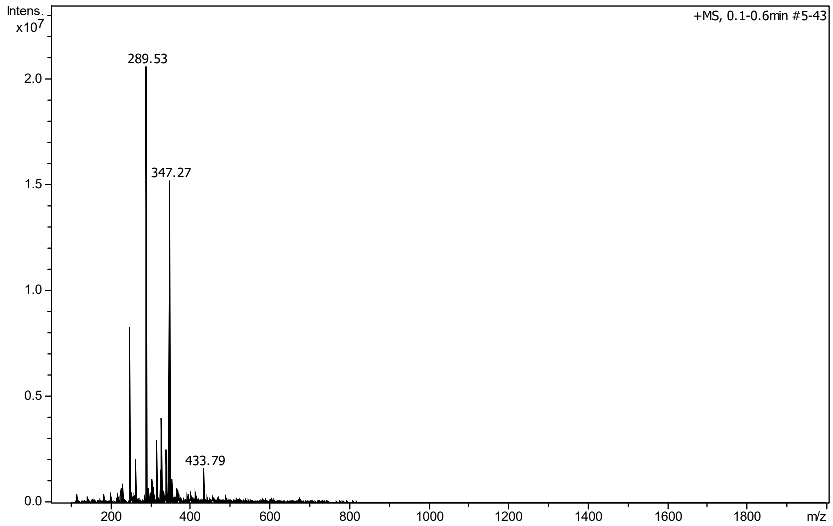

Figure S41. MS Spectrum of Cy5-Arg8**.** The identity of the peptide conjugate was determined using Bruker Amazon SL (Germany) ion trap mass spectrometer. The samples are dissolved in water-acetonitrile (50:50) with 0.1% acetic acid.

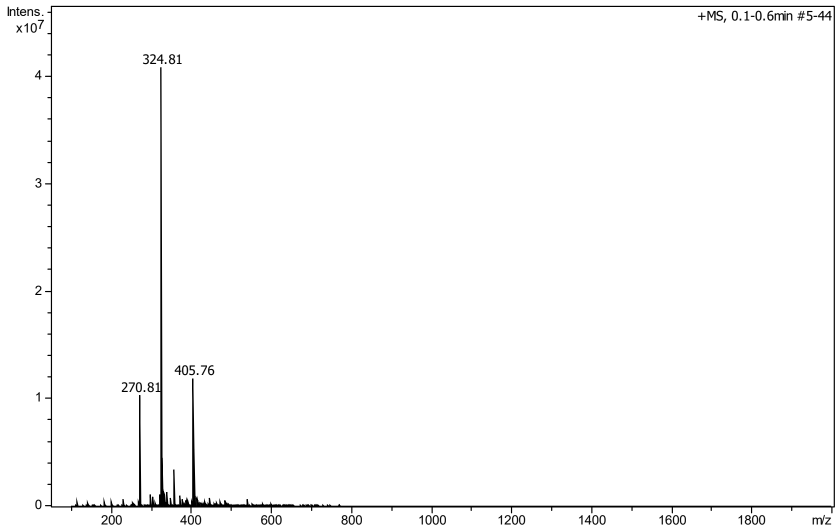

Figure S42. MS Spectrum of Dabcyl-AMBA-RRRRK(Cy5). The identity of the peptide conjugate was determined using Bruker Amazon SL (Germany) ion trap mass spectrometer. The samples are dissolved in water-acetonitrile (50:50) with 0.1% acetic acid.

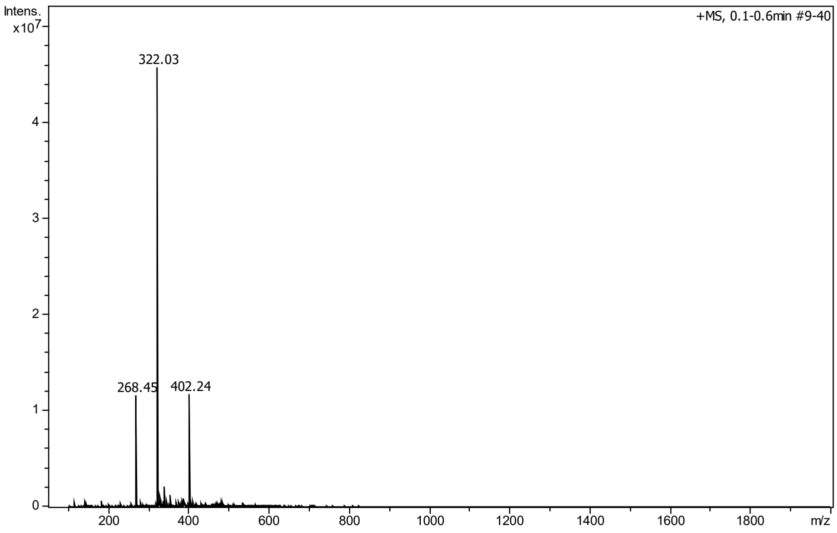

Figure S43. MS Spectrum of Dabcyl-PABA-RRRRK(Cy5). The identity of the peptide conjugate was determined using Bruker Amazon SL (Germany) ion trap mass spectrometer. The samples are dissolved in water-acetonitrile (50:50) with 0.1% acetic acid.

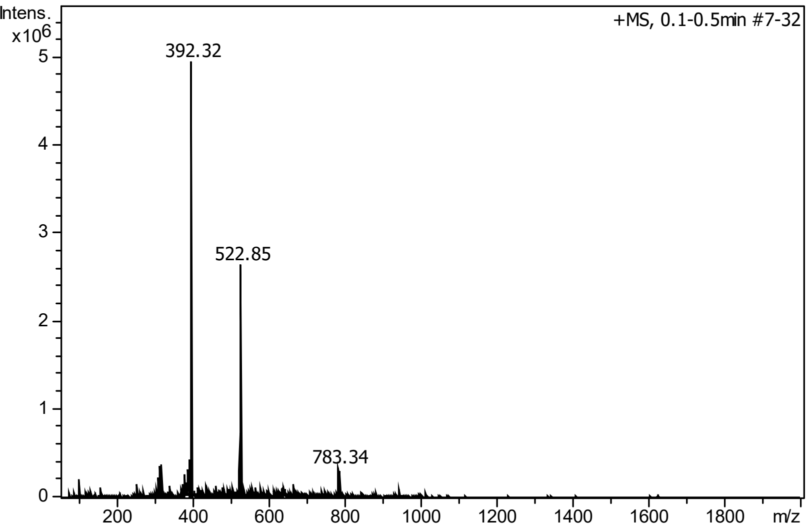

Figure S44. MS Spectrum of Dabcyl-RRWRRK(Cf). The identity of the peptide conjugate was determined using Bruker Daltonics Esquire 3000 plus (Germany) ion trap mass spectrometer. The samples are dissolved in water-acetonitrile (50:50) with 0.1% acetic acid.
